# A unified view to Brønsted acidity scales: do we need solvated protons?†
†Electronic supplementary information (ESI) available: Detailed description of the equilibria, activities, anchoring approach and experiments. See DOI: 10.1039/c7sc01424d



**DOI:** 10.1039/c7sc01424d

**Published:** 2017-08-07

**Authors:** Eno Paenurk, Karl Kaupmees, Daniel Himmel, Agnes Kütt, Ivari Kaljurand, Ilmar A. Koppel, Ingo Krossing, Ivo Leito

**Affiliations:** a Institute of Chemistry , University of Tartu , Ravila 14a Str , 50411 Tartu , Estonia . Email: ivo.leito@ut.ee; b Institut für Anorganische und Analytische Chemie and Freiburger Materialforschungs-zentrum (FMF) , Albert-Ludwigs-Universität Freiburg , Albertstr. 21 , 79104 Freiburg , Germany . Email: krossing@uni-freiburg.de

## Abstract

The most comprehensive solvent acidity scale spanning 28 orders of magnitude of acidity was measured in the low-polarity solvent 1,2-dichloroethane (DCE). Its experimental core is linked to the unified acidity scale (pH_abs_) in an unprecedented and generalized approach only based on experimental values. This enables future measurements of acid strengths and acidity adjustments in low polarity solvents. The scale was cross-validated computationally. The purely experimental and computational data agree very well. The DCE scale includes 87 buffer systems with  values between –13.0 and +15.4, *i.e.* similar to water at hypothetical and extreme pH values of –13.0 to +15.4. Unusually, such high acidities in DCE are not realized *via* solvated protons, but rather through strongly acidic molecules able to directly donate their proton, even to weak bases dissolved in the solution. Thus, in all examined cases, not a single solvated proton is present in one liter of DCE.

## Abstract 2

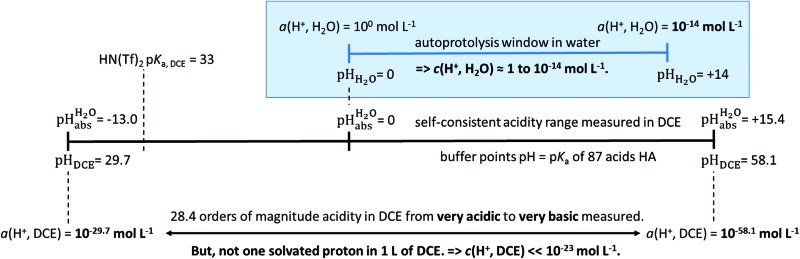
The most comprehensive solvent acidity scale spanning 28 orders of magnitude of acidity was measured in the low-polarity solvent 1,2-dichloroethane (DCE).

## Introduction

The proton is undoubtedly one of the most important particles in the universe. The most prominent phenomenon associated with it – acidity‡
‡We exclusively use the term “acidity” for proton acidity according to Arrhenius, Brønsted and Lowry.^1–3^

^1–3^ – is so important to nature that living organisms even have a sense for it. Acid–base reactions are ubiquitous, but are mainly investigated in polar and/or “protic” solvents containing ionizable protons at concentrations that allow for a direct measurement, *e.g.* by conductivity. Similarly, superacid chemistry is mainly done in highly polar, protic media like HF/SbF_5_ mixtures or the legendary “magic acid” HSO_3_F/SbF_5_.^4^ This overall polarity of a solvent medium physically arises from the hydrogen bond donor and acceptor capacity, polarizability and dipole moment of its molecules and the dipole density that allow for stabilizing interactions with the dissolved ions, thus lowering their chemical potential (partial molar free energy, *i.e.* rate of change of the free energy of the system with respect to the change in the number of molecules of the respective species that are added to the system). If the medium is non-polar, the solvation of ions, including H^+^, is weak and their chemical potential is high.^5^ Thus, paradoxically, the highest acidities should be achievable in an environment that is as non-polar as possible. In agreement with this, numerous – *e.g.* acid-catalyzed – chemical reactions are carried out both in the lab as well as on an industrial scale in non- to weakly polar aprotic solvents with relative dielectric constants *ε*_r_ roughly between 2 and 14 (such as alkanes, substituted arenes, ethers or haloalkanes).^6^ Azeotropic dehydrations in non-polar toluene with toluenesulfonic acid present a popular example. Thus, acid chemistry in non-polar and non-basic solvents has been used in practice for decades. However, wouldn’t it be fundamentally interesting to learn about the medium acidity of this and other popular mixtures in quantitative terms, in order to rigorously compare reaction conditions in one solvent to another?

Towards this general goal of the thermodynamically sound evaluation of acidity, we have now derived a general approach to establish unified acidities^5^ in solvent media only on the basis of experimental values. This fundamental development is applied here to the exemplarily selected low-polarity solvent 1,2-dichloroethane (DCE, *ε*_r_ = 10.36 (ref. 7)). Due to its low basicity, but sufficient polarity to dissolve polar and ionic compounds at measurable concentrations/activities, DCE is a suitable solvent for studying acids and also superacids. The scale covers 28 orders of magnitude of acidity and is linked to the unified acidity scale (pH_abs_ scale).^5^ It is based on our earlier work^8^ on the high acidities in DCE (stronger acids than picric acid, spanning about 15 orders of magnitude). Finally, we support the experimental results by a cross-validation based on quantum-chemical calculations with consideration of solvation effects by the SMD,^9^ rCCC,^10^ and COSMO-RS^11,12^ models.

## Definitions and concepts

Acidity in non-aqueous media, and especially the comparison of acidity across media or even phase boundaries, is not common knowledge and quantitative views remain sparse. Therefore, we start with essential definitions and concepts.

### pH and the chemical potential of the proton

pH is the most popular measure for medium acidity. In its standard definition, pH_S_ = –log(*a*(H^+^, S)), it is a measure for the solvated proton’s chemical potential *μ*_abs_(H^+^, S) in the solvent/medium S. The molecular acidity of an acid HA in a solvent S is described by the equilibrium (1),

1 HA_(solv.)_ ⇄ A_(solv.)_^–^ + H_(solv.)_^+^

and the available activity *a*(H^+^, S) of solvated protons H_(solv.)_^+^ formally determines the equilibrium pH_S_ value in S, *i.e.* its acidity in medium S (and limited to medium S). Decreasing the pH of a solution by one unit increases the proton’s chemical potential and thus the solution’s acidity by *RT* ln 10 = 5.71 kJ mol^–1^ at standard conditions (25 °C, 1 bar). The intrinsic molecular acidity of HA in S is quantitatively given by its acidity constant *K*_a,S_ (eqn (1)), used as the negative decadic logarithm p*K*_a,S_.2
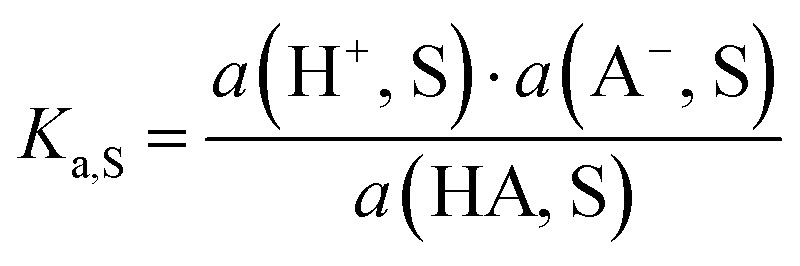



Importantly, the pH_S_ values as defined above – using reference states in medium S – are bound to the medium S and cannot be used to compare acidities in different solvents/media.

### Unified acidity scale

Following the seminal work of Bartmess,^13^ we introduced the ideal proton gas at one bar as a unified – and medium-independent – reference state for acidity and as a thermodynamic zero point.^5^ The chemical potential of this reference state “proton gas” is set to 
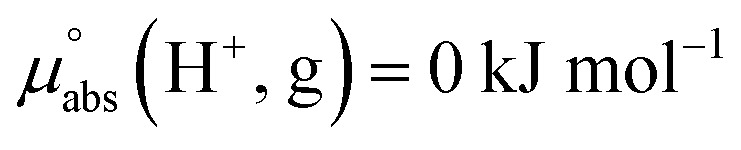
 and pH_abs_ = 0. The independence of this reference state from any medium allows for a unified comparison of acidities in terms of chemical potentials or the corresponding pH_abs_ values in different media on an absolute basis. For example, water with a pH_water_ of 0 has a pH_abs_ of 193.5 (with the published Gibbs hydration energy of the gaseous proton (Δ_solv._ *G*°(H^+^, H_2_O) = –1105 kJ mol^–1^).^14–16^ In order to express pH_abs_ in a more familiar way, it is useful to shift it by 193.5 units, to obtain so-called 
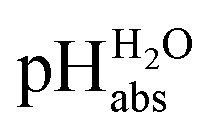
 values 
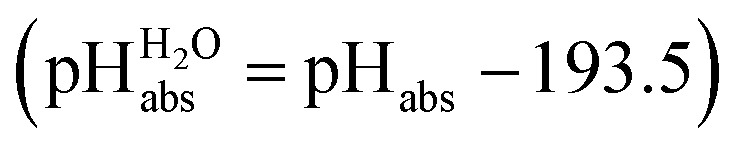
,^17^ which are a direct continuation of aqueous pH values and identical to them in water. This means that the chemical potential of the proton in any solvent/medium with 
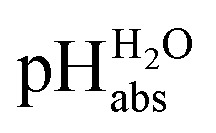
 5 is the same as pH_water_ 5.

### Acidity measurements in low polarity media

Acidity measurements in low polarity media become increasingly difficult and only limited examples are known.^8^ An acidity scale was even measured in the very non-polar *n*-heptane,^18^ but it is obvious that protons solvated by heptane itself are not present in solution, as protonated *n*-heptane would decompose with the loss of H_2_ or an alkane and rearrange instantly to a more stable branched tertiary carbenium ion. With the commonly used notion that acidity relies on dissolved ions, it needs acids with a p*K*_a,S_ in a given solvent of less than 12 to obtain micro-molar ion concentrations that can be easily measured. In low polarity media, the choice of such acids is very limited. Only extreme acids are able to generate ions in low polarity media to a measurable/isolable extent. A drastic example is H[HCB_11_F_11_] that even protonates the very non-polar medium liquid carbon dioxide giving the salt H(CO_2_)_2_^+^[HCB_11_F_11_]^–^.^19^ Since low polarity solvents weakly solvate ions, the extent of ion–ion interactions and aggregation increases drastically in such media. Therefore, effects like ion-pairing and others have to be evaluated and correction schemes have to be applied to obtain the “real acidity” data (*cf.* ESI Sections 2 and 3†). In addition, traces of basic impurities like water compete with the solvent base for the proton and therefore have a dramatic effect on the acid dissociation, *e.g.* by formation of (also further solvated) H_3_O^+^. This effect is evaluated in a later section.

### Superacidity

Superacidity refers to the highest acidity and can be used in relation to media or molecules. Thus, we differentiate between two principal types of superacidity:

#### Medium superacidity

Medium superacidity was initially defined by Gillespie as the acidity of a medium that is “stronger than sulfuric acid”.^20^ In the context of the unified acidity scale, a superacidic medium is one where the chemical potential of the proton lies higher than that in 100% sulfuric acid. Although the Hammett^21^ function *H*_0_ is most frequently used to assess superacidity,^22^ it does not directly express the proton’s chemical potential and thus should not be considered a thermodynamic acidity scale (see ref. 5 and 10).§
§From principal thermodynamic considerations, the *H*_0_ curve follows d*H*_0_ = –*RT* ln 10 × (d*μ*(H^+^) – d*μ*(BH^+^) + d*μ*(B)) and is “contaminated” by the indicator base system’s chemical potential change. In the unified acidity scale, the absolute chemical potential of the proton in neutral¶
¶“Neutral” means that the pH_H_2_SO_4__ is half of the p*K* of autoprotolysis. It is commonly addressed as “100% H_2_SO_4_”. liquid H_2_SO_4_ was assessed as the threshold for superacidity: if a medium S has *μ*_abs_(H^+^, S) < *μ*_abs_(H^+^, H_2_SO_4_ (l)) or –975 kJ mol^–1^, it is superacidic.^5,10^ This corresponds to a 
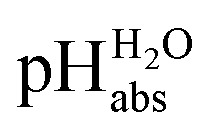
 below –22.4.‖
‖This indicates that the acidity level rise from water to pure sulfuric acid is about ten orders of magnitude higher (!) than the *H*_0_ value of pure sulfuric acid of –11.9 would suggest. As the main reason for this, we assume that the desolvation of the proton is accompanied by a desolvation of the protonated indicator base, which dampens the *H*_0_ curve.


#### Molecular superacidity

Molecular superacidity refers to an acidic molecule/molecular ion, which, in a given solvent/medium S, is a stronger acid than sulfuric acid, *i.e.* it has a lower p*K*_a,S_ value in this solvent/medium than sulfuric acid. In the gas phase,^23^ a superacidic molecule or molecular ion has a lower gas phase acidity (GA) value than the gaseous H_2_SO_4_ molecule.

## The equilibrium acidity scale in DCE

Buffer solutions are a superior means to measure the self-consistent equilibrium acidity scale in DCE, both for reasons of the measurable concentrations of ions as well as the stability of the protochemical potential. The buffers used herein, *i.e.* mixtures of acids and bases, keep the acidity at an approximately constant value and allow for the reliable and reproducible measurement of the chemical potential. In the simplest case, a 1 : 1 mixture of an acid with a salt of its conjugate base buffers at the buffer point pH_S_ = p*K*_a,S_. To construct a self-consistent ladder of relative acidities, protonation equilibria between a large number of acid pairs (involving all acids on the scale) have to be evaluated^24^ in the solvent S according to equilibrium (3),

3 HA_1 (solv.)_ + A–2 (solv.) ⇄ A–1 (solv.) + HA_2 (solv.)_

where HA_1_ and HA_2_ are the two acids. Their relative acidity difference Δp*K*_a,S_ in solvent S can therefore be calculated as follows (4):4
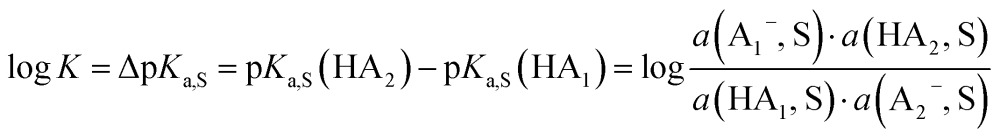



However, the direct measurement in DCE gives values slightly differing from Δp*K*_a,S_ (eqn (4)) due to ion-pairing effects. Those are denoted as Δp*K*_ip_ (ip stands for ion-pairing). In this work, the acidity scale^8^ in DCE is presented, composed of a total of 226 Δp*K*_ip_ measurements between 87 acids. For the construction of the scale, the sum of the squares of the differences between the experimental Δp*K*_ip_ values and the differences between the assigned p*K*_ip,rel_ values was minimized. Taking picric acid as a reference acid by arbitrarily assigning a value of 0.0 to its p*K*_ip,rel_ value gave a self-consistent p*K*_ip,rel_ ladder (Table 2).^8^ It should be noted that in principle every acid could be taken as the reference acid without affecting the accuracy of the procedure. Using picric acid is rather traditional (it is also used as the reference acid in acetonitrile^24^). To obtain the Δp*K*_a,DCE_ values, all p*K*_ip,rel_ values were corrected (by up to 0.51 units, see ESI, Section 3†) for the logarithmic difference of the ion-pair dissociation constants Δp*K*_d_ of the acids under study using the Fuoss model as described in the methods section and the ESI (Section 2†).^25^ Thus, one obtains the corrected thermodynamic p*K*_a,rel_ values with respect to picric acid (Table 2). The compiled self-consistent acidity scale in Table 2 spans 28.4 orders of magnitude. The overall reliability of the logarithmic acidity constant values was checked using the consistency standard deviation.^8^ For both p*K*_ip,rel_ and p*K*_a,rel_ values, it is 0.04 log units or 0.23 kJ mol^–1^, which can be considered very good for a low polarity solvent.

## Anchoring the DCE scale to 
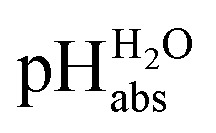
 values

The construction of a pH_abs_ scale hitherto required in all cases the Gibbs solvation energy for at least one ion, for example the hydration energetics of the gaseous proton (Δ_solv._ *G*°(H^+^, H_2_O) = –1105 kJ mol^–1^).^14–16^ This value is not directly accessible by experimental methods, and was obtained by the extrapolation of the hydration thermodynamics of gaseous ion–water clusters to the bulk solvent. According to the authors, it is tainted with an error bar of at least ±8 kJ mol^–1^ or ±1.4 pH units! Advantageously, in the 
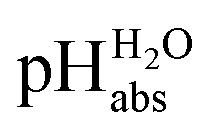
 scale proposed herein, large single ion Gibbs solvation energies can be removed or replaced by single ion Gibbs transfer energies, which are typically lower by 1–2 orders of magnitude. This reduces the overall uncertainty in the chemical potential assessment. Still, for partitioning the chemical potential of dissolved ionic compounds into single ion values, so-called extrathermodynamic assumptions have to be introduced. The most used assumption by far is the so-called “TATB assumption”.^26^ It assumes that when transferring the salt tetraphenylarsonium-tetraphenylborate (TATB) from one medium to the other, the chemical potential difference of both ions is about the same between the solvents and the Gibbs transfer energy of each ion just amounts to half of the measurable sum.^27^ Although this assumption is disputed,^28^ any error or difference between these reference ions will systematically add to the single ion quantities, so that the overall structure of the 
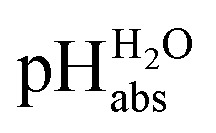
 scale will remain intact.

### General approach

Neither the medium p*K*_a,DCE_ of acids nor pH_DCE_ values are experimentally accessible (see below). Despite this, we can construct a thermodynamically consistent 
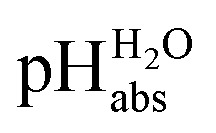
 scale using single ion Gibbs transfer energies from water to DCE *via* suitable Born–Fajans–Haber cycles (BFHCs, see ESI Section 6 for details†). The only acid in Table 2 for which sufficient experimental data for such a connection to aqueous pH exists is HCl. Fortunately, the Gibbs solvation energies of single ions cancel out in this calculation or can be replaced by one single ion Gibbs transfer energy, namely that of the chloride ion. According to these calculations, detailed in the ESI in Section 5,† a 1 : 1 HCl/Cl^–^ buffer mixture in DCE has an acidity that corresponds to pH 2.5 in water. With the knowledge that HCl has a p*K*_a,rel_ of 0.2 *vs.* picric acid (Table 2), we obtain the simply applicable universal formula (5) that is valid for all our measured acids:5
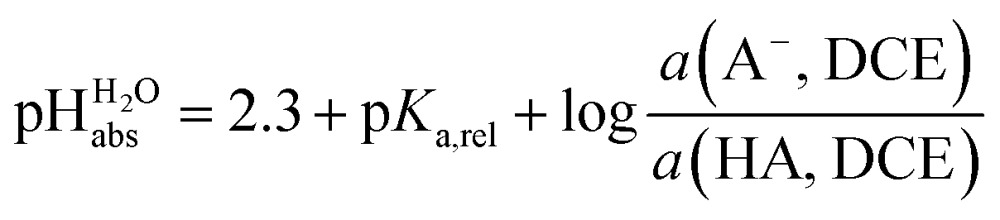



Using formula (5), we obtain a measured 
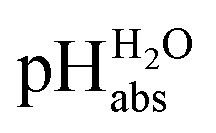
 range in DCE between +15.4 for the weakest acid 9-COOMe-fluorene and –13.0 for the strongest acid CN-TCNP in 1 : 1 (neutral acid : acid anion) buffer solutions. Such 1 : 1 buffer solutions correspond to the so-called buffer point (BP) and the corresponding 
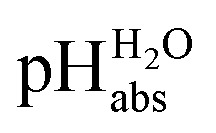
 can be termed as 
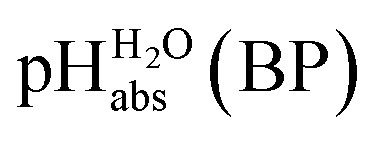
 and calculated *via*eqn (6).6
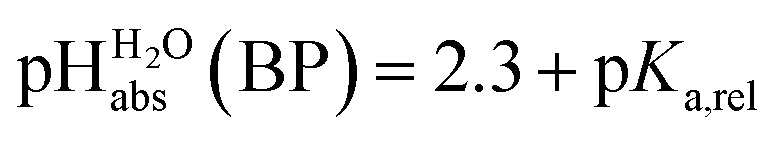



The 
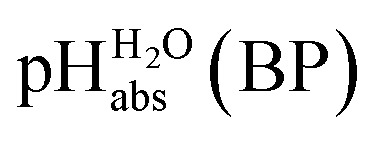
 values for all of the acids are presented in Table 2. Using the acids from Table 2, DCE solutions of well-defined unified acidity, *i.e.* 
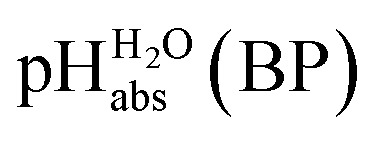
 values, can be prepared on the basis of our data together with eqn (5) and (6) by any first-year chemist. For higher ion concentrations, a correction scheme for non-ideality (Debye–Hückel effects) is deposited in the ESI, Section 7.†

## Cross validation: quantum-chemical solvation models *vs.* experimental data

Several terms sum up to the overall Gibbs solvation energy as calculated by quantum chemistry. For small ions and molecules, the electrostatic part is very sensitive to the chosen calotte radii or isodensity surface and dominates the overall solvation thermodynamics. For large ions and molecules, the van-der-Waals interaction as well as the cavity energy becomes increasingly important. Thus, here we decided to use the medium sized HNTf_2_/NTf_2_^–^ system for model building. An evaluation of the favorable performance of this system by comparing calculated (rCCC model) and experimental p*K*_a_s of HNTf_2_ in MeCN or DMSO was published.^29^ For validation, the p*K*_a,rel_ (HNTf_2_, DCE) of –12.0 from Table 2 was set into eqn (6). We obtain 
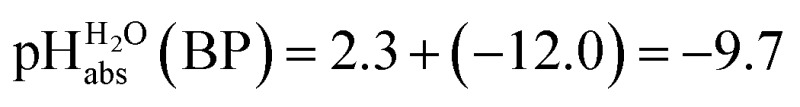
 for HNTf_2_ in DCE at the buffer point BP (*i.e.* pH_S_ = p*K*_a,S_). As described above, the main error source in this value is the uncertainty of Δ_tr_ *G*°(Cl^–^, DCE → H_2_O), obtained with the TATB assumption. Eqn (7)7
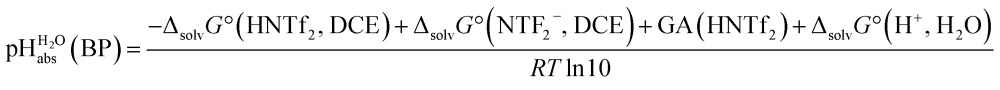
can be used for a cross validation with the solvation models. For this, we augmented our published calculated rCCC^10^ and SMD^30^ data set with COSMO-RS^11,12^ solvation calculations (see ESI Section 14 for details†). By combining the experimental Δ_solv._ *G*°(H^+^, H_2_O) (–1105 kJ mol^–1^) and the experimental gas phase acidity (1199 kJ mol^–1^)^31^ of HNTf_2_ with the quantum-chemically calculated Gibbs solvation energies of HNTf_2_ and NTf_2_^–^, we obtained the validation data in Table 1.

**Table 1:** Cross-validation data of the experimentally assessed relation 5 *versus* rCCC, SMD and COSMO-RS derived values (eqn (7)). Δ_solv._ *G*° values are in kJ mol^–1^

Model	Δ_solv._ *G*° (HNTf_2_, DCE)	Δ_solv._ *G*° (NTf_2_^–^, DCE)	p*K*_a_, DCE (HNTf_2_)	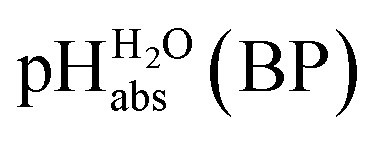
rCCC	–26	–177	32.9	–9.9
SMD	–15	–148	32.6	–6.7
COSMO-RS	–20	–176	33.1	–10.7
Exp.	—	—	—	–9.7

Pleasingly and supporting our experimental findings, the calculated 
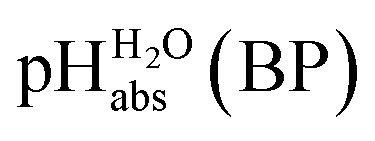
 values between –6.7 and –10.7 collected in Table 1, and obtained with the different quantum-chemical solvation models, agree within –1.0/+3.0 pH units to the experimental one at –9.7. This corresponds to –5.7/+17.1 kJ mol^–1^ at standard conditions. It should be noted that, in addition to the error in the calculated Gibbs solvation energies, the Δ_solv._ *G*°(H^+^, H_2_O) value has an estimated error bar^14–16^ of ±8 kJ mol^–1^ or ±1.4 pH units. In the end, it cannot be decided whether the experimental or one of the quantum chemically calculated 
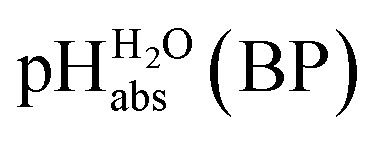
 values is more accurate.

### Does one need solvated protons for protonation of bases?

One may be surprised that neither our experimental 
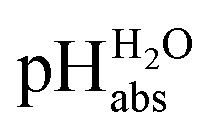
 values (Tables 1 and 2) nor our 
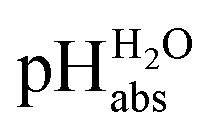
 calculations based on quantum-chemical solvation models (Table 1) require knowledge of the medium pH_DCE_ or any information about the solution thermodynamics of the solvated proton in DCE. The reason is that the proton bound in neutral HNTf_2_ is in equilibrium with the solvated proton in DCE. Thus, in chemical equilibrium, the chemical potentials of the neutral acid and of the dissociated proton as well as the acid anion according to the relation (8)

8 HNTf_2_(DCE) ⇄ H^+^(DCE) + [NTf_2_]^–^(DCE)

are equal. Therefore, although the actual concentration of the solvated protons H^+^(DCE) is vanishingly small in DCE, the solution contains a manifold of bound protons at the same chemical potential in the neutral acid moelcules! This means that, if a basic molecule is immersed into the solution, it will be protonated by the neutral, non-dissociated acid present – here HNTf_2_(DCE) – and not by the hardly existing solvated protons!

**Table 2:** Acidity scale in 1,2-dichloroethane; see the text for in-depth explanations of the data

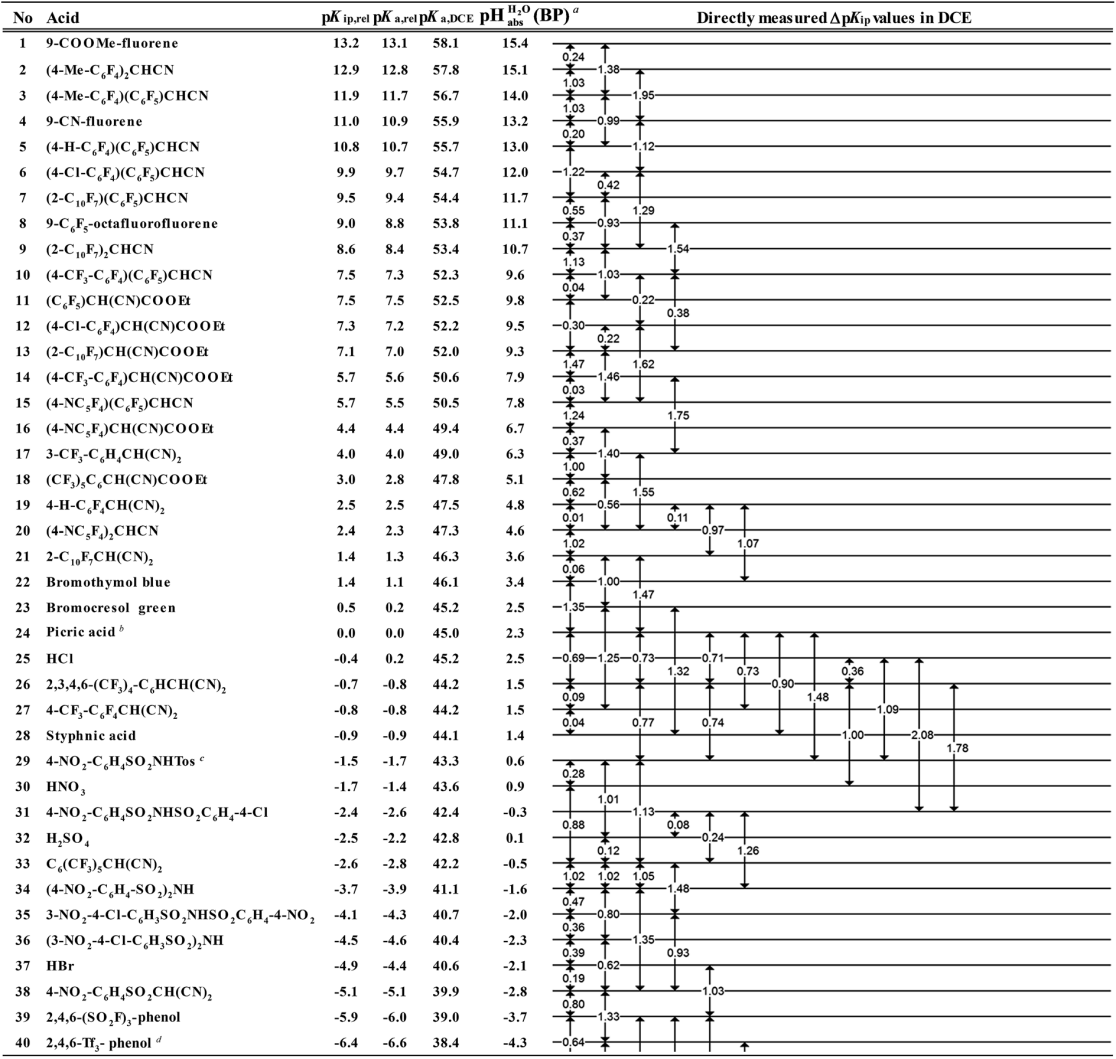
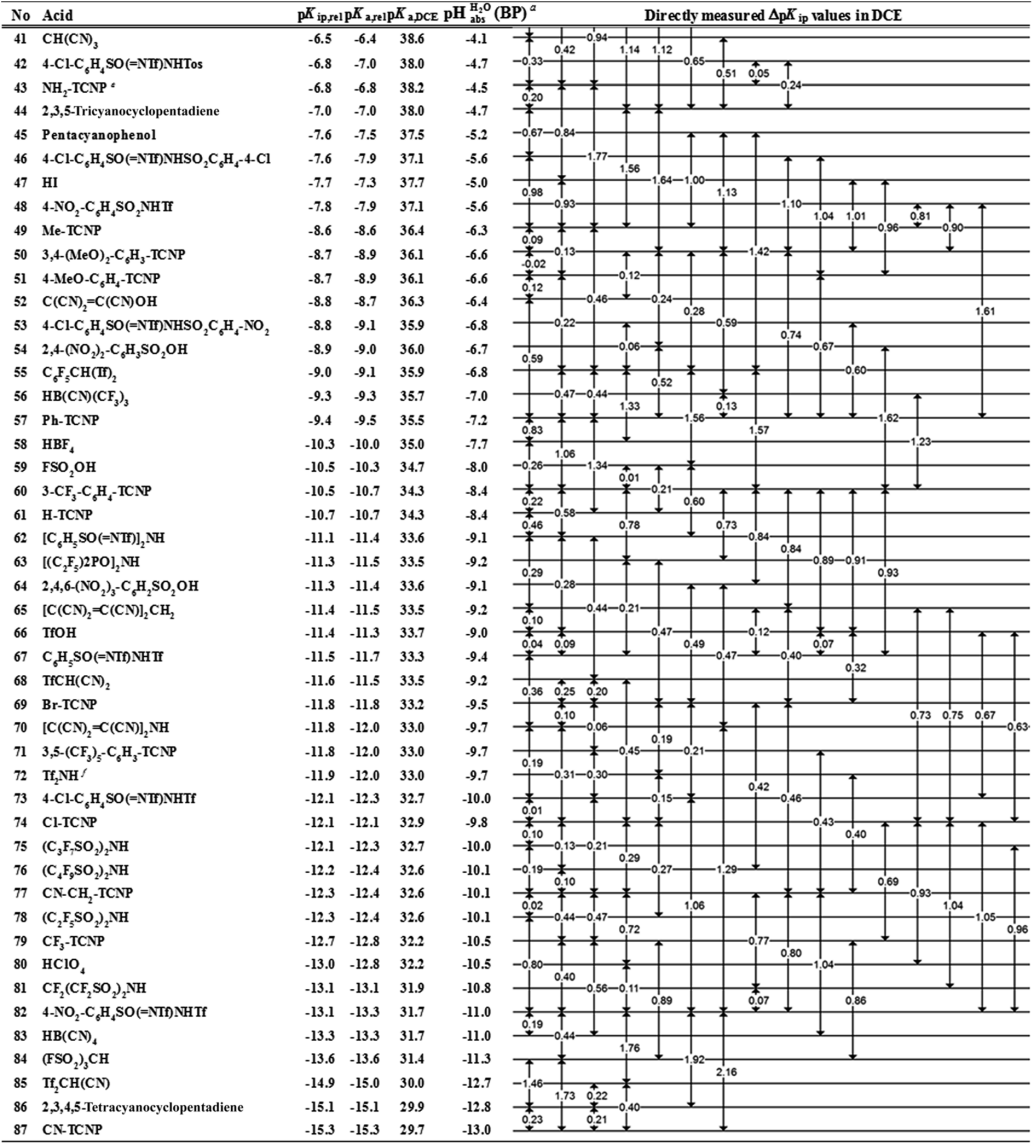

^*a*^Estimates of absolute acidities in terms of aqueous pH of 1 : 1 HA/A^–^ buffer solution in DCE of the respective acid.

^*b*^p*K*_ip,rel_ value of picric acid is arbitrarily set to 0.

^*c*^Tos represents the 4-Me-C_6_H_4_SO_2_-group.

^*d*^Tf represents the CF_3_SO_2_-group.

^*e*^X-TCNP represents 2-X-1,1,3,3-tetracyanopropene.

^*f*^Reference acid for the p*K*_a,DCE_ values with a computational p*K*_a,DCE_ value of 33.

### Is it possible to obtain true medium p*K*_a_ values in DCE?

In order to transfer the obtained relative p*K*_a,rel_ values, with respect to picric acid, to medium p*K*_a,DCE_ values, we need the reliable medium p*K*_a,DCE_ of at least one of the acids we measured, as an anchor point. Our calculation of the p*K*_a,DCE_ value of HNTf_2_ in DCE gave rather robust values all rounding to 33, irrespective of the solvation model used (range: 32.6–33.1, Table 1).^30^ Even taking the lowest p*K*_a,DCE_ of 32.6, this would imply that in a 0.01 M solution of HNTf_2_ in pure DCE only 5 × 10^–18^ mol acid per liter is dissociated. This is unmeasurable! An additional problem arises from the low basicity of DCE: tiny traces of more basic impurities, especially water, have an enormous influence on acid dissociation and make medium p*K*_a,DCE_ determinations (nearly) impossible. Even well dried DCE (around 0.15 ppm) still contains around 10^–5^ mol L^–1^ of water. A rough SMD calculation gave a p*K*_a,DCE_ value of 13.6 for the H_3_O^+^ cation in DCE (see ESI Sections 7 and 13 for details†). According to the law of mass action this means that even at a 2.5 × 10^–14^ mol L^–1^ (!) concentration of water, 50% of all solvated protons would be attached to water molecules and not to DCE. Even in buffered solutions, we see no chance for directly measuring p*K*_a,DCE_ or pH_DCE_ values in this case. Therefore, and in order to obtain estimates of true medium p*K*_a,DCE_ values (eqn (2)), the scale was reconstructed with the corrected p*K*_a,rel_ values and the computationally assessed medium p*K*_a,DCE_ value of HNTf_2_. Since all values in Table 1 round to 33, we used this value as an anchor point (entry 72 in Table 2).

## The DCE acidity scale: explanation and discussion of the entries in Table 2

The columns p*K*_ip,rel_, p*K*_a,DCE_ and 
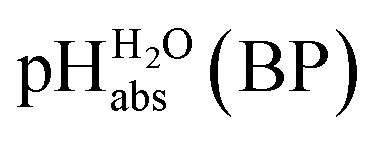
 of Table 2 illustrate the different levels of complexity in data treatment and the corresponding depth of information that can be obtained. In short:

• p*K*_ip,rel_ – accurately measured experimental and strictly ion-pair (molecular) acidities of compounds relative to picric acid in DCE. The relative values are reliable, but their absolute magnitudes are arbitrary. The relative values are robust within the medium, as are approximations used in other nonpolar media, and depend less on impurities than the absolute values and are thus useful for comparing acids, *e.g.* to rationalize synthesis conditions or design electrochemical cells within DCE.

• p*K*_a,rel_ – estimate of the relative (to picric acid) p*K*_a_ value obtained from p*K*_ip,rel_ *via* correction for ion-pairing (using the Fuoss equation^25^).

• p*K*_a,DCE_ – the common measure of (molecular) acidity of compounds, *i.e.* ionic acidity values as defined by the negative decadic logarithm of eqn (1). In non-polar media p*K*_a,S_ values are very difficult or impossible to measure directly, but can be derived from p*K*_a,rel_ by anchoring to a robust computational value (here: p*K*_a,DCE_(HNTf_2_) = 33). The values are dependent on the medium and thus are strictly non-comparable between different media.

• 
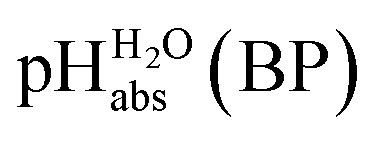
 – medium acidity (as opposed to molecular acidity) of a 1 : 1 buffer solution expressed in relation to the aqueous pH scale. It is derived experimentally from the p*K*_ip,rel_ values in Table 2 at the buffer point (BP), corrected to relative p*K*_a,rel_ and anchored to the 
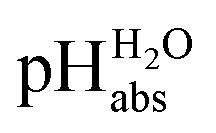
 scale *via* the experimental procedure delineated above. The values correspond to the thermodynamic proton activity in water at the same pH values, and are comparable between different media.

### Do acids exist that allow for direct p*K*_a_ determination in DCE?

In the course of direct p*K*_a_ measurements, when increasing the acidity of the solution, traces of H_2_O, rather than DCE molecules, will be protonated. With the SMD-calculated medium p*K*_a,DCE_(H_3_O^+^) of 13.6, 0.01 M of an acid HA (as a function of p*K*_a,DCE_(HA)) and in DCE with 10^–5^ M water contamination (0.14 ppm), only acids with p*K*_a,DCE_ values below 6.7 are suitable for direct medium p*K*_a,DCE_ measurements. This is 23 orders of magnitude more acidic than the strongest acid CN-TCNP included within Table 2 (entry 87; p*K*_a,DCE_ 29.7). However, do acids with such p*K*_a_ values exist in DCE? Halogenated derivatives of *closo*-dodecaborane or 1-carba-undecaborane acids may have such acidities.^30^ Our computational estimates of their medium p*K*_a,DCE_ values in DCE are –0.1 for H_2_[B_12_F_12_] and –1.6 for H[CB_11_F_12_], which means that they should be fully dissociated in DCE solutions! The strongest acid prepared to date is H[HCB_11_F_11_],^32^ which is only weaker by 21 kJ mol^–1^ ^33^ than H[CB_11_F_12_], and thus it is also expected to be sufficiently dissociated in DCE.**
**However, a further problem arises from the fact that even the slightly weaker carborane acids like H[HCB_11_Cl_11_] and H[HCB_11_Br_6_H_5_]^37,38^ as well as the H_2_[B_12_X_12_] (X = Cl, Br) acids^39^ are known to decompose many solvents including dichloromethane^37^ and thus most probably also DCE by elimination of HCl and formation of carbocations.


### Can we reach superacidity in DCE?

#### Medium superacidity

From the p*K*_a,rel_ of sulfuric acid (–2.2), we can calculate a 
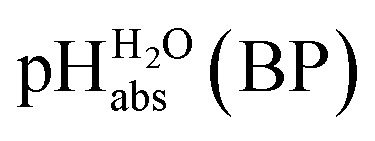
 of 0.1 for a sulfuric acid buffer in DCE, which is very far off the –22.4 we suggest for neutral sulfuric acid as a medium. The main reason is that a conjugate acid anion (here: [HSO_4_]^–^) is much less solvated and thus much more basic in DCE than in protic solvents. Even our strongest acid buffer 
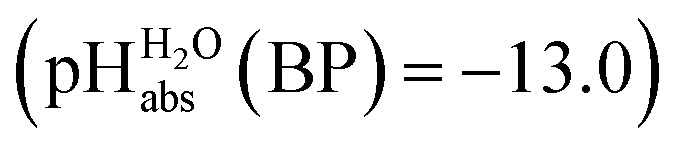
, including the molecular superacid CN-TCNP, is more than 9 orders of magnitude away from showing medium superacidity as defined above. But, with the above delineated calculated p*K*_a,DCE_ values of the carborane acids, it is clear that medium superacidity may be reached in DCE, yet only with special and difficult to prepare acids, and is awaiting experimental realization.

#### Molecular superacidity

By contrast, 55 entries in Table 2 are molecular superacids in DCE holding a lower medium p*K*_a,DCE_ value than sulfuric acid.

## Solvent effects on acidity: a comparison to the gas phase and MeCN

Many acids studied in this work were investigated in acetonitrile (MeCN) and in the gas phase (Table S1 in the ESI†). p*K*_a,DCE_ and p*K*_a,MeCN_ are very well correlated (Fig. S3, ESI†), as described by (9):

9 p*K*_a,DCE_ = 1.08 p*K*_a,MeCN_ + 33.0, *s*(slope) = 0.02; *s*(intercept) = 0.2; *n* = 44; *R*^2^ = 0.992; *S* = 0.6

This indirectly supports the quality of the results and provides a convenient tool to predict the acidities of strong acids in MeCN, which cannot be directly measured in that solvent. As expected, DCE is about 8% more differentiating than MeCN. The intercept at 33 is just the difference between the standard Gibbs solvation energies of the proton in the two media, *i.e.* in MeCN (1058 kJ mol^–1^) and DCE (869 kJ mol^–1^). This difference of 189 kJ mol^–1^ converted into the log-scale by division through 5.71 kJ mol^–1^ gives 33.

### Why are the DCE and GA values poorly correlated?

Given the inertness of DCE as a solvent, one could expect a good correlation between the acidities in DCE and the gas phase. The reality is very different. The correlation across all acid families is next to non-existent. It thus turns out that even such a low-polarity and inert solvent as DCE is by its influence on acid ionization much more similar to polar aprotic solvents (MeCN) than to the gas phase. Better correlations are obtained when acidities within families are compared (see ESI, Fig. S4†). The poor correlation can be rationalized from a thermodynamic cycle (S-BFHC 3 in ESI†) for the protonation equilibrium between two acids. Thus, the relation between medium p*K*_a,S_ values and GA values can be derived as:10
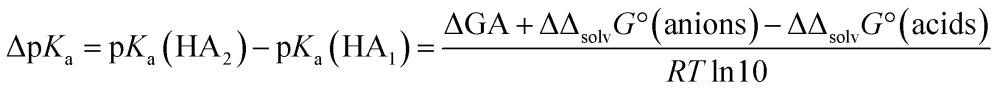



A linear correlation would require that the Gibbs solvation energy difference between a certain acid and its anion is identical for all acids. Obviously, this is not at all the case.

## Conclusions

**Fig. 1:**
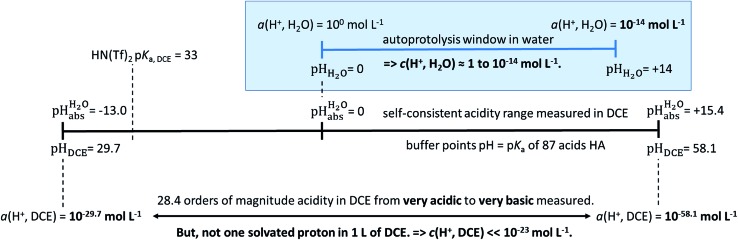
Relation between the medium acidities in water and DCE and the 
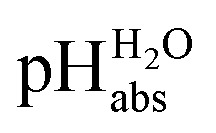
 scale and the limiting activities of the solvated proton in these media.

Using acids from Table 2, buffer solutions of a well-defined composition can be prepared spanning an acidity range of over 28 pH units (or 160 kJ mol^–1^), which is double the pH window of water. Considering an acid/base catalyzed reaction, this means an acceleration or slow-down factor of 10^28^. This corresponds to the difference between one millisecond and the age of the universe! It is as yet unclear if medium p*K*_a,DCE_ and pH_DCE_ values can ever be directly measured in DCE. Despite this, experimental 
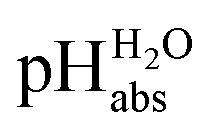
 values were established in this work that describe the solvated proton’s thermodynamics without any knowledge of the proton’s specific solvation and its activity in DCE. The quality of the derived 
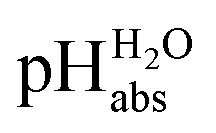
 values mainly depends on the quality of the extrathermodynamic assumption (in our case the TATB assumption) and the accuracy of the so obtained single ion transfer thermodynamics. This approach is general and can be transferred to any solvent S, given that at least one acid is known for which the transfer energies from water to S exist for all particles. The general relation between aqueous acidities and the range of our buffer acidities measured is shown in Fig. 1. One can easily see that with our used buffer systems, the protolytic window of water is by far exceeded, if compared on the unified acidity scale. However, according to our assessed medium p*K*_a,DCE_ values, even in our most acidic buffer system CN-TCNP, the proton concentration of its buffer in DCE of 10^–29.7^ mol L^–1^ is much less than one proton per liter. The latter would correspond to 1.6 × 10^–24^ mol L^–1^. This clearly shows that for measurable acid thermodynamics, solvated protons do not need to exist in the medium. Rather, the bound proton in the solvated neutral acid, being in equilibrium with the ionic solvated proton, has the same chemical potential and accounts for the protonation event. However, especially for non-polar media and in unbuffered solutions, impurities may determine the pH_S_. Thus, chemical reactions influenced by acidity may proceed in an unpredictable way, if not run in a carefully selected buffer, for example selected from the 87 systems collected in Table 2. The good correlations between the p*K*_a,DCE_ values and the corresponding p*K*_a_ values in MeCN, as well as in heptane and DMSO (Fig. S7 and S8 in the ESI, respectively†), suggest that the values in Table 2 can, in principle, be reliably transferred to other organic solvents.

### Quantum chemical methods

Quantum chemical calculations on the SMD model were done with the same procedure as in ref. 30. rCCC calculations were done with the same procedure as in ref. 10. COSMO-RS^11,12^ input files (.cosmo and .energy) were created with the COSMO^34^ module of the Turbomole^35^ program system according to the “BP_TZVPD_FINE_C30_1501.ctd” formalism. The COSMOTherm^36^ program was used to calculate COSMO-RS Gibbs solvation energies.

## Conflicts of interest

There are no conflicts to declare.

## List of abbreviations/compendium

Unified acidity: It allows a unified view to acidity over phase and medium boundaries. It is set absolute with the pH_abs_ value with respect to the reference state proton gas
p*K*_a,S_: Negative decadic logarithm of the medium acidity constant in the solvent S
p*K*_a,DCE_: Negative decadic logarithm of the medium acidity constant in the solvent DCE
p*K*_a,H_2_O_: Negative decadic logarithm of the medium acidity constant in the solvent H_2_O
p*K*_ip,rel_: Negative decadic logarithm of the relative (to picric acid) acidity constant of the acid–titrant base ion pair (here the solvent is DCE, unless stated otherwise)
p*K*_a,rel_: Directly measured p*K*_ip,rel_ corrected for ion-pairing effects according to the Fuoss-model to a true relative (to picric acid) acidity constant (here the solvent is DCE, unless stated otherwise)
*a*(H^+^, S): Activity of the solvated proton in solvent/medium S
*a*(H^+^, DCE): Activity of the solvated proton in solvent DCE
*a*(H^+^, H_2_O): Activity of the solvated proton in solvent H_2_O
pH_S_: Negative decadic logarithm of the activity of the solvated proton *a*(H^+^, S) in solvent/medium S
pH_DCE_: Negative decadic logarithm of the activity of the solvated proton *a*(H^+^, DCE) in solvent DCE
pH_H_2_O_: Negative decadic logarithm of the activity of the solvated proton *a*(H^+^, H_2_O) in solvent H_2_O
S: The liquid medium the acidity is investigated in. It may be a molecular solvent like DCE or a strong acid itself

## References

[cit1] Arrhenius S. A. (1887). Z. Phys. Chem..

[cit2] Brønsted J. N. (1923). Recl. Trav. Chim. Pays-Bas.

[cit3] Lowry T. M. (1923). J. Chem. Technol. Biotechnol..

[cit4] Olah G. A., Superacid chemistry, Wiley, Hoboken, N.J., 2nd edn, 2009.

[cit5] Himmel D., Goll S. K., Leito I., Krossing I. (2010). Angew. Chem., Int. Ed..

[cit6] (a) Wypych G., Handbook of solvents, ChemTec Publ, Toronto, 2001.

[cit7] Reichardt C. and Welton T., Solvents and solvent effects in organic chemistry, Wiley-VCH, Weinheim, Germany, 4th edn, 2011.

[cit8] Kütt A., Rodima T., Saame J., Raamat E., Maemets V., Kaljurand I., Koppel I. A., Garlyauskayte R. Y., Yagupolskii Y. L., Yagupolskii L. M., Bernhardt E., Willner H., Leito I. (2011). J. Org. Chem..

[cit9] Marenich A. V., Cramer C. J., Truhlar D. G. (2009). J. Phys. Chem. B.

[cit10] Himmel D., Goll S. K., Leito I., Krossing I. (2011). Chem.–Eur. J..

[cit11] Klamt A. (1995). J. Phys. Chem..

[cit12] Klamt A., Jonas V., Bürger T., Lohrenz J. C. W. (1998). J. Phys. Chem. A.

[cit13] Bartmess J. E. (1994). J. Phys. Chem..

[cit14] Tissandier M. D., Cowen K. A., Feng W. Y., Gundlach E., Cohen M. H., Earhart A. D., Coe J. V., Tuttle T. R. (1998). J. Phys. Chem. A.

[cit15] Tissandier M. D., Cowen K. A., Feng W. Y., Gundlach E., Cohen M. H., Earhart A. D., Tuttle T. R., Coe J. V. (1998). J. Phys. Chem. A.

[cit16] Kelly C. P., Cramer C. J., Truhlar D. G. (2006). J. Phys. Chem. B.

[cit17] Suu A., Jalukse L., Liigand J., Kruve A., Himmel D., Krossing I., Roses M., Leito I. (2015). Anal. Chem..

[cit18] Rõõm E.-I., Kaljurand I., Leito I., Rodima T., Koppel I. A., Vlasov V. M. (2003). J. Org. Chem..

[cit19] Cummings S., Hratchian H. P., Reed C. A. (2016). Angew. Chem., Int. Ed..

[cit20] Gillespie R. J. and Peel T. E., in Advances in physical organic chemistry, ed. V. Gold and D. Bethell, Elsevier Academic Press, London, 2003, vol. 9, pp. 1–24.

[cit21] Hammett L. P., Deyrup A. J. (1932). J. Am. Chem. Soc..

[cit22] Jorgenson M. J., Hartter D. R. (1963). J. Am. Chem. Soc..

[cit23] Koppel I. A., Taft R. W., Anvia F., Zhu S.-Z., Hu L.-Q., Sung K.-S., DesMarteau D. D., Yagupolskii L. M., Yagupolskii Y. L. (1994). J. Am. Chem. Soc..

[cit24] Kütt A., Leito I., Kaljurand I., Soovali L., Vlasov V. M., Yagupolskii L. M., Koppel I. A. (2006). J. Org. Chem..

[cit25] Abdur-Rashid K., Fong T. P., Greaves B., Gusev D. G., Hinman J. G., Landau S. E., Lough A. J., Morris R. H. (2000). J. Am. Chem. Soc..

[cit26] Alexander R., Parker A. J. (1967). J. Am. Chem. Soc..

[cit27] Marcus Y., Kamlet M. J., Taft R. W. (1988). J. Phys. Chem..

[cit28] Schurhammer R., Wipff G. (2000). J. Mol. Struct.: THEOCHEM.

[cit29] Kögel J. F., Linder T., Schroder F. G., Sundermeyer J., Goll S. K., Himmel D., Krossing I., Kutt K., Saame J., Leito I. (2015). Chem.–Eur. J..

[cit30] Lipping L., Leito I., Koppel I., Krossing I., Himmel D., Koppel I. A. (2015). J. Phys. Chem. A.

[cit31] Leito I., Raamat E., Kutt A., Saame J., Kipper K., Koppel I. A., Koppel I., Zhang M., Mishima M., Yagupolskii L. M., Garlyauskayte R. Y., Filatov A. A. (2009). J. Phys. Chem. A.

[cit32] Küppers T., Bernhardt E., Eujen R., Willner H., Lehmann C. W. (2007). Angew. Chem., Int. Ed..

[cit33] Lipping L., Leito I., Koppel I., Koppel I. A. (2009). J. Phys. Chem. A.

[cit34] Klamt A., Schüürmann G. (1993). J. Chem. Soc., Perkin Trans. 2.

[cit35] Ahlrichs R., Bär M., Häser M., Horn H., Kölmel C. (1989). Chem. Phys. Lett..

[cit36] (a) Eckert F. and Klamt A., COSMOTherm, COSMOtherm, C3.0, release 1501, COSMOlogic GmbH & Co KG, 2015, http://www.cosmologic.de.

[cit37] Reed C. A. (2010). Acc. Chem. Res..

[cit38] Juhasz M., Hoffmann S., Stoyanov E., Kim K.-C., Reed C. A. (2004). Angew. Chem., Int. Ed..

[cit39] Avelar A., Tham F. S., Reed C. A. (2009). Angew. Chem., Int. Ed..

