# Supplementary material for: A unified view to Brønsted acidity scales: do we need solvated protons?
Source: Chem Sci. 2017 Aug 7;8(10):6964–73. doi: 10.1039/c7sc01424d (PMC5642146; doi:10.1039/c7sc01424d)
Supplement: Supplementary file 1 [file SC-008-C7SC01424D-s001.pdf]

Electronic Supplementary Material (ESI) for Chemical Science.
This journal is © The Royal Society of Chemistry 2017

Supplementary Information to

A Unified View to Brønsted Acidity Scales: Do we need Solvated Protons?

Eno Paenurk,[a)] Karl Kaupmees,[a)] Daniel Himmel,[b)] Agnes Kütt,[a)] Ivari Kaljurand,[a)] Ilmar A. Koppel,[a)] Ingo Krossing,*[,b)] Ivo Leito*[,a)]

[a)] Institute of Chemistry, University of Tartu, Ravila 14a Str, 50411 Tartu, Estonia. Correspondence regarding to acidity measurements should be addressed to I.L. (email: ivo.leito@ut.ee)

[b)] Institut für Anorganische und Analytische Chemie and Freiburger Materialforschungs-zentrum (FMF), Albert-Ludwigs-Universität Freiburg, Albertstr. 21, 79104 Freiburg, Germany. Correspondence regarding to unified acidity should be addressed to I.K. (email: krossing@uni-freiburg.de).

Table of Contents

1. Detailed description of acid-base equilibrium 2
2. Ion-pair acidities and free ion acidities 2
3. Relation between p$K_{ip,rel}$ and p$K_{a,rel}$ values 3
4. Activities and concentrations 4
5. Other equilibria in DCE 4
6. Anchoring approach 5
7. Excursus: Estimation of non-ideality 6
8. Influence of Water on Equilibrium Ion Concentrations 7
9. Compatibility of the expansion 9
10. Limitation of DCE in the high pKa end of the scale 9
11. Solvent and substituent effects on acidity 11
12. Experimental 18
13. Synthesis of compound 18 19
14. Details on the quantum chemical calculations 21

1. Detailed description of acid-base equilibrium. The acidity of a substance in a solvent is realized by the transfer of proton from the acid (HA) to the solvent molecule (S) that acts as a base. This reaction is a combination of equilibria:

$$(\mathrm{HA})_\mathrm{s} + (\mathrm{:S})_\mathrm{s} \overset{K_1}{\rightleftarrows} (\mathrm{AH\cdots:S})_\mathrm{s} \overset{K_2}{\rightleftarrows} (\mathrm{A:^-\cdots HS^+})_\mathrm{s} \text{ or } (\mathrm{A:^-HS^+})_\mathrm{s} \overset{K_3}{\rightleftarrows} (\mathrm{A:^-||HS^+})_\mathrm{s} \overset{K_4}{\rightleftarrows}$$

$$\overset{K_4}{\rightleftarrows} (\mathrm{A:^-})_\mathrm{s} + (\mathrm{HS^+})_\mathrm{s} \qquad \text{(S1)}$$

The subscript s denotes solvation of the corresponding species. The equilibrium constants as given in Equation (S1) describe the following reactions[1]:

- K_1 – formation of a hydrogen-bonded complex
- K_2 – proton transfer within the complex from the acid to the solvent. Depending on the properties of HA and S, either a new hydrogen-bonded complex or an ion-pair is formed. High dielectric permittivity and strong Lewis acidic/basic properties of the solvent favor the forward reaction.
- K_3 – formation of a solvent-separated ion-pair in which the ions are separated by a thin layer of solvent molecules. Large ions with delocalized charge tend to form contact ion-pairs and this reaction is thus favored by ions of smaller size. Specific solvation of the ions also favors the forward reaction.
- K_4 – dissociation, i.e. formation of "free" solvated ions. This requires sufficiently high relative permittivity of the solvent ($\varepsilon_r > 15$).

These equilibrium constants enter the simplified acidity constant as $K_\mathrm{a}=a(\mathrm{S})\cdot K_1\cdot K_2\cdot K_3\cdot K_4$, in which the activity of the solvent is absorbed into the K_a value by definition[1]. The simplified reaction as given by Equation (1) in the main text describes acidity in a polar solvent. In nonpolar solvents such as DCE, the reaction may halt at equilibrium K_2 or K_3, depending on the acid. The resulting equilibrium then describes acidity until formation of ion-pairs, i.e. ion-pair acidity.

2. Ion-pair acidities and free ion acidities. The similarity between ion-pair acidities (p$K_\mathrm{ip,rel}$) and free ion acidities (p$K_\mathrm{a,rel}$), both expressed relative to picric acid, was established via calculations of ion-pair dissociation constants. The first step is calculation of difference of ion-pair dissociation constants by the formula derived from the Fuoss model.[2] This formula works satisfactorily if one type of ion-pair is present (e.g. acid anions with the protonated base), there are no specific interactions between ions and the ions are close to spherical. These conditions can be considered satisfactorily met in this work. Details about the method for ΔpK_d calculations in this work are presented in section 3.

$$\Delta\mathrm{p}K_\mathrm{d} = -C\cdot\left(\frac{1}{r_{\mathrm{A_1^-}}+r_{\mathrm{HB^+}}}-\frac{1}{r_{\mathrm{A_2^-}}+r_{\mathrm{HB^+}}}\right)+3\log\left(\frac{r_{\mathrm{A_2^-}}+r_{\mathrm{HB^+}}}{r_{\mathrm{A_1^-}}+r_{\mathrm{HB^+}}}\right) \qquad \text{(S2)}$$

In (S2), r is the radius of the corresponding ion in Å and C is a combined constant that has a value of 23.5 Å for DCE at 25 °C.

The approximated relative ionic acidity* then follows as (S3).[3]

$$\Delta \mathrm{p}K_{\mathrm{a}} = \Delta \mathrm{p}K_{\mathrm{ip}} - \log \frac{K_{\mathrm{d}}^{\mathrm{HB^+A_2^-}}}{K_{\mathrm{d}}^{\mathrm{HB^+A_1^-}}} = \Delta \mathrm{p}K_{\mathrm{ip}} + \Delta \mathrm{p}K_{\mathrm{d}} \qquad \text{(S3)}$$

The $\mathrm{p}K_{\mathrm{ip,rel}}$ values, differently from $\mathrm{p}K_{\mathrm{a,rel}}$ values, are influenced by ion pairing between the acid anion and the cation of the titrant base. The large size of the cation decreases the influence and its dependence on the anion size, but it is still not negligible and is expected to be the strongest for small anions (cf. Section 3). Therefore the estimates of $\mathrm{p}K_{\mathrm{a}}$ values are more rigorous in describing acidity. The error bar of this correction could be around 0.1 $\mathrm{p}K_{\mathrm{a}}$ units. However, it is largely a systematic effect, meaning that the relative acidities ($\Delta\mathrm{p}K_{\mathrm{a}}$ values) of acids will have much lower error bars.

3. Relation between $\mathrm{p}K_{\mathrm{ip,rel}}$ and $\mathrm{p}K_{\mathrm{a,rel}}$ values. It was previously assumed that $\mathrm{p}K_{\mathrm{a,rel}} \approx \mathrm{p}K_{\mathrm{ip,rel}}$ in DCE medium.[4] In this paper, this assumption was further studied by direct calculation of $\mathrm{p}K_{\mathrm{a,rel}}$ values using the procedure delineated in the previous section and used in previous works.[2,3]

Approximate relative acidity $\Delta\mathrm{p}K_{\mathrm{a}}$ values were calculated according to Equations S2 and S3. Firstly, ion geometries were optimized with semi-empirical PM3 method with Spartan'08 software.[5] Ion radii were determined from optimized ion geometries by two methods. One method used computed ion volume to calculate radius from the sphere volume formula. The other method involved measuring the sides of an imaginary cuboid, inside which a space-filling model of an ion would exactly fit. Those side lengths were averaged and divided by two. The resulting radii from these two methods were averaged for every ion.

The results are shown in Figure S1. For nearly 90% of the acids, the difference of $\mathrm{p}K_{\mathrm{ip,rel}}$ and $\mathrm{p}K_{\mathrm{a,rel}}$ is less than 0.25 units. However, for acids with very large or very small anions, the difference exceeds 0.3 units and goes up to 0.51 units in the case of **25** (hydrogen chloride). The smaller the anion of an acid, the lower the $\mathrm{p}K_{\mathrm{ip,rel}}$ compared to $\mathrm{p}K_{\mathrm{a,rel}}$.

---

* Such $\mathrm{p}K_{\mathrm{a}}$ estimates have been denoted as $\mathrm{p}K_{\alpha}$ in similar works on bases in tetrahydrofuran.[2,3]

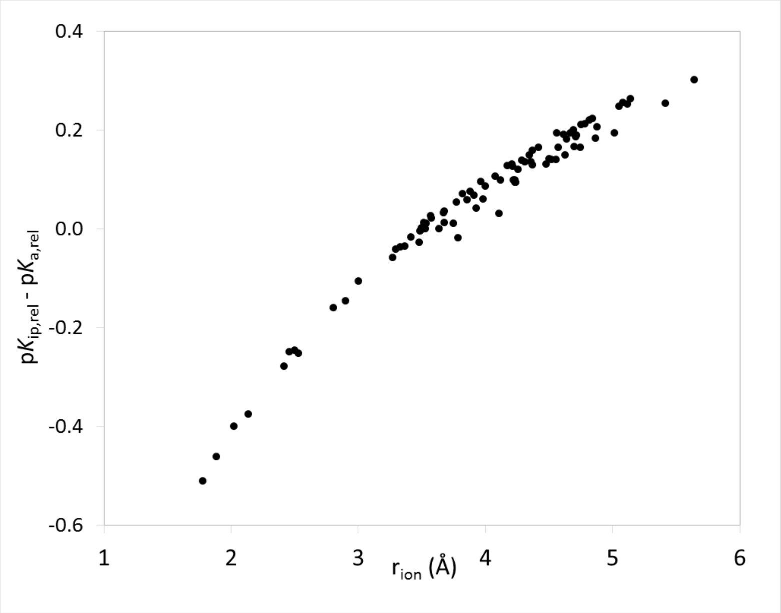

Figure S1. Dependence of the difference of p$K_{ip,rel}$ and p$K_{a,rel}$ values on anion radius.

4. Activities and concentrations. For acid-base species with identical charge types, the ratio of activity coefficients of neutral and charged species can be assumed to be the same in diluted solutions, as proposed by Hammett and Deyrup.[6] This assumption allows using concentrations that can be measured by spectrophotometry, instead of activities in Equation 4 in the paper. The assumption has been successfully used in similarly constructed acidity scales[1,4,7,8].

5. Other equilibria in DCE. In addition to ion-pairing, other equilibria can complicate investigating acid-base equilibria in DCE. Conjugation processes of neutral and charged species is the most probable of such equilibria.[4] The conjugation of the charged and neutral species of an acid is called homoconjugation[1]:

$$A_1^- + HA_1 \xrightleftharpoons{K_{homo}} [A_1\cdots HA_1]^- \quad (S4)$$

In case of conjugation of neutral and charged species of different acids, the process is called heteroconjugation[1]:

$$A_1^- + HA_2 \xrightleftharpoons{K_{hetero}} [A_1\cdots HA_2]^- \quad (S5)$$

These processes, and also formation of larger aggregates, are most likely to occur in weakly solvating solvents, such as DCE, with compounds that are good hydrogen bond donors or acceptors.[8] These equilibria can be suppressed by using dilute solutions[1] as used in this work. In addition, the backbone of the acidity scale was constructed using acids with extensively delocalized charge in corresponding anions. This further decreases the likelihood of the presence of unwanted equilibria.

6. Anchoring approach. We start with the aqueous hydrogen chloride electrolysis reaction (S-BFHC 1):

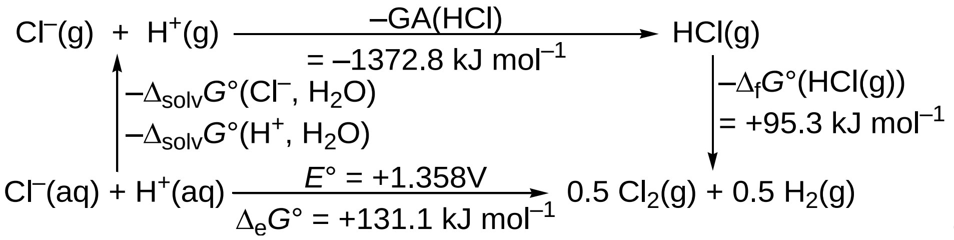

(S-BFHC 1)

Using well established $E°(Cl_2/Cl^-, H_2O)$ = 1.358 V,[9] $\Delta_f G°(HCl(g))$ = –95.3 kJ mol^{-1},[9] and GA(HCl) = 1372.8 kJ mol^{-1},[10] one can derive expression (S6) for $\Delta_{solv}G°(Cl^-, H_2O)$.

$$\Delta_{solv}G°(Cl^-, H_2O) = -FE°(Cl_2/Cl^-, H_2O) - \Delta_{solv}G°(H^+, H_2O) - GA(HCl) - \Delta_f G°(HCl(g))$$

$$= -1408.6\ kJmol^{-1} - \Delta_{solv}G°(H^+, H_2O) \qquad (S6)$$

Now let us consider the hypothetical BFHC 2, where the dissolved hydrogen chloride releases its proton directly into the gas phase:

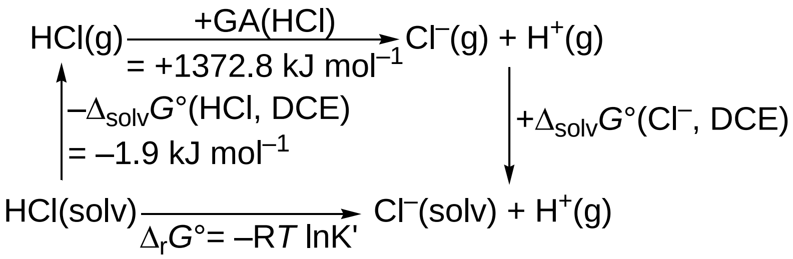

(S-BFHC 2)

The equilibrium constant for this reaction is *K*' (S7)

$$K' = \frac{a(Cl^-(solv) \times p(H^+, g)}{a(HCl(solv)) \times 1bar} \qquad (S7)$$

Note that $p(H^+, g)$ directly transforms to the absolute pH_{abs} value. Resolving the law of mass action in logarithmic form gives (S8):

$$pH_{abs} = \frac{-\Delta_{solv}G°(HCl, DCE) + GA(HCl) + \Delta_{solv}G°(Cl^-, DCE)}{RT\ln 10} + \log\frac{a(Cl^-(solv))}{a(HCl(solv))} \quad (S8)$$

From the equilibrium solubility of HCl gas in DCE,[11] $\Delta_{solv}G°(HCl, DCE)$= +1.9 kJ mol^{-1} can be derived. In this formula, we can substitute (S9)

$$\Delta_{solv}G°(Cl^-, DCE) = \Delta_{solv}G°(Cl^-, H_2O) + \Delta_{tr}G°(Cl^-, H_2O \rightarrow DCE) \qquad (S9)$$

Inserting the expression for $\Delta_{solv}G°(Cl^-, H_2O)$ derived from the aqueous BFHC 1 yields (S10):

$$\mathrm{pH_{abs}} = \frac{-\Delta_{\mathrm{solv}}G^\circ(\mathrm{HCl,DCE})+\mathrm{GA(HCl)}+\Delta_{\mathrm{tr}}G^\circ(\mathrm{Cl^-,H_2O\rightarrow DCE})}{\mathrm{R}T\ln 10}$$

$$+\frac{-\mathrm{F}E^\circ(\mathrm{Cl_2/Cl^-,H_2O})-\Delta_{\mathrm{solv}}G^\circ(\mathrm{H^+,H_2O})-\mathrm{GA(HCl)}-\Delta_{\mathrm{f}}G^\circ(\mathrm{HCl(g)})}{\mathrm{R}T\ln 10}+\log\frac{a(\mathrm{Cl^-(solv)})}{a(\mathrm{HCl(solv)})} \qquad \text{(S10)}$$

Note that in formula (S10) GA(HCl) cancels out. Inserting this formula (S10) into the definition for $\mathrm{pH_{abs}^{H_2O}}$ (S11)

$$\mathrm{pH_{abs}^{H_2O}} = \mathrm{pH_{abs}} + \frac{\Delta_{\mathrm{solv}}G^\circ(\mathrm{H^+,H_2O})}{\mathrm{R}T\ln 10} \qquad \text{(S11)}$$

also $\Delta_{\mathrm{solv}}G^\circ(\mathrm{H^+,H_2O})$ cancels out. One obtains (S12):

$$\mathrm{pH_{abs}^{H_2O}} = \frac{1}{\mathrm{R}T\ln 10}\left(-\mathrm{F}E^\circ(\mathrm{Cl_2/Cl^-,H_2O}) - \Delta_{\mathrm{solv}}G^\circ(\mathrm{HCl,DCE}) - \Delta_{\mathrm{f}}G^\circ(\mathrm{HCl(g)}) + \Delta_{\mathrm{tr}}G^\circ(\mathrm{Cl^-,H_2O\rightarrow DCE})\right) + \log\frac{a(\mathrm{Cl^-(solv)})}{a(\mathrm{HCl(solv)})} \qquad \text{(S12)}$$

$$= 2.5 + \log\frac{a(\mathrm{Cl^-(solv)})}{a(\mathrm{HCl(solv)})}$$ (with $\Delta_{\mathrm{tr}}G^\circ(\mathrm{Cl^-, DCE\rightarrow H_2O}) = +52\ \mathrm{kJ\ mol^{-1}}$ [12])

Thus, a 1:1 $\mathrm{HCl/Cl^-}$ buffer mixture in DCE has an acidity that corresponds to pH 2.5 in water. With the knowledge that HCl has a $\mathrm{p}K_{\mathrm{a,rel}}$ of 0.2 vs. picric acid, we obtain the simply applicable universal formula (S13) that is valid for all our measured acids:

$$\mathrm{pH_{abs}^{H_2O}} = 2.3 + \mathrm{p}K_{\mathrm{a,rel}}(\mathrm{HA}) + \log\frac{a(\mathrm{A^-(solv)})}{a(\mathrm{HA(solv)})} \qquad \text{(S13)}$$

7. Excursus: Estimation of non-ideality. The formula (5) in the article contains the thermodynamically needed activities instead of easier to measure concentrations. Assuming ideal solution behavior for neutral compounds and applying the extended Debye-Hückel law[13] for ion activity coefficients, we can introduce an activity coefficient corrected formula that uses the simpler to obtain concentrations. The so-called Debye length L_{D} is calculated from the ion strength $I = {}^1\!/_2 \sum_i c_i z_i^2$ via (S14):

$$L_{\mathrm{D}} = \sqrt{\frac{\varepsilon_{\mathrm{r}} kT}{2\mathrm{N_A}\mathrm{e}^2 I}} \qquad \text{(S14)}$$

The Debye length can be interpreted as the radius of a contrary charged "ion cloud" that forms around every ion decreasing its activity. Debye-Hückel theory is of course only valid, if this Debye length is much larger than the ion radius itself. For ion strengths of 10^{-4} M, 10^{-3} M, and 10^{-2} M, the Debye lengths are 11.1 nm, 3.5 nm, and 1.1 nm, respectively. From this, the activity coefficients of the ions are calculated via (S15):

$$\ln f_i = -\frac{\mathrm{e}^2}{8\pi\varepsilon_0\varepsilon_{\mathrm{r}}kT}\times\frac{L_D^{-1}}{1+L_D^{-1}r_i} \qquad \text{(S15)}$$

Assuming a ion radius of r_i of 0.3 nm, the plot for the decadic log f as shown in Fig. S2 is obtained. Further assuming ideal solvation behavior of the neutral acid in DCE, a concentration dependent formula is obtained (S16):

$$\mathrm{pH}_{\mathrm{abs}}^{\mathrm{H_2O}} = 2.3 + \mathrm{p}K_{\mathrm{a,rel}} + \log f + \log\frac{[\mathrm{A^-(solv)}]}{[\mathrm{HA(solv)}]} \quad (S16)$$

Overall, we estimate a moderate deviation of less than one pH unit for ion concentrations lower than 10^{-2} M due to non-ideality.

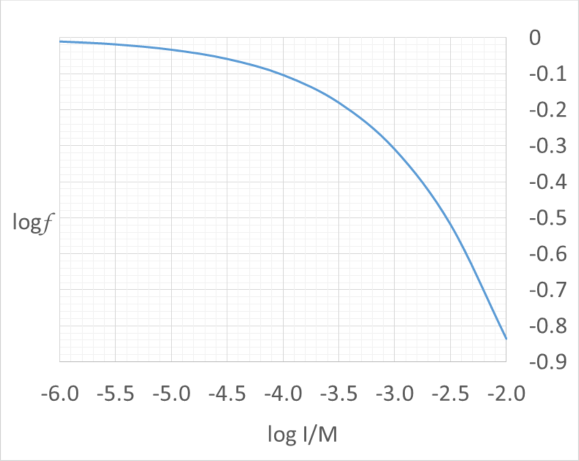

Figure S2. Dependence of the ion activity coefficient from the ionic strength in DCE.

It should be stressed here that the Debye-Hückel theory contains approximations that become increasingly unreliable with ion concentrations above ~10^{-3} M. At ion concentrations below 10^{-4} M, log f deviates by less than 0.1 pH unit and essentially ideal behavior can be assumed. For our following considerations, we assume ideal behavior for simplicity.

8. Influence of Water on Equilibrium Ion Concentrations. To get a clue of the influence of water (or other impurities) on the equilibrium ion concentrations of unbuffered acid solutions, we made model calculations for DCE containing 10^{-5} M water contamination, assuming a medium pK_a(H_3O^+) of 13.6, and 0.01 M of an acid as a function of the medium pK_a of the acid. The ion equilibrium concentrations are shown in Figure S3. For comparison, also the H^+ equilibrium concentration calculated for pure DCE (free of any water) is shown.

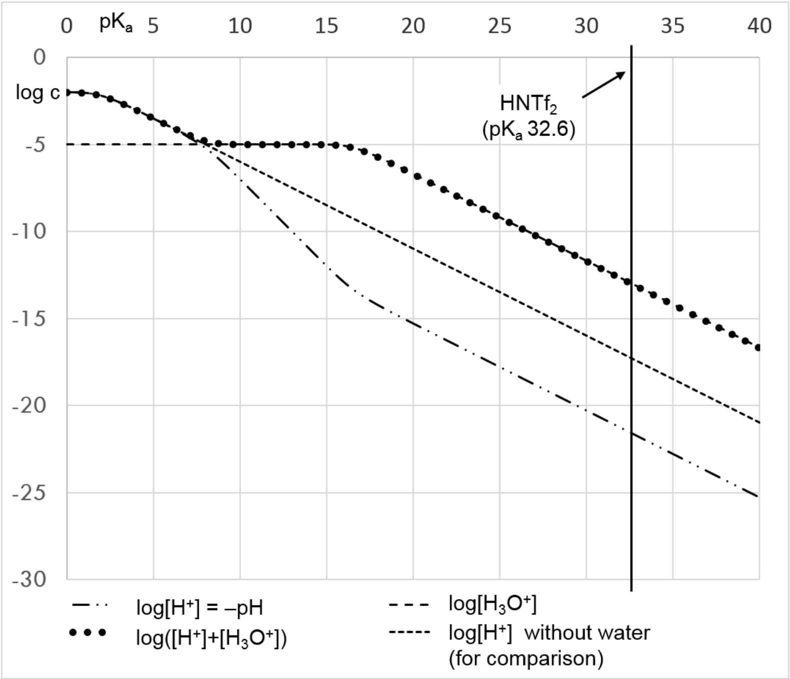

Figure S3. Equilibrium ion concentrations in DCE containing 10^{-5} M water and 0.01 M acid as a function of the pK_a of the acid.

The vertical line shows the situation in the case of 0.01 M $HNTf_2$. As discussed in the paper, in water-free DCE there would be a proton concentration of $10^{-17.3}$ M, *i.e.* a pH of 17.3. The water contamination shifts the pH to 21.6. This means that due to the water impurity, the chemical potential of the proton is lowered by 4.3 kcal mol^{-1}. In an electrochemical measurement, a reversible hydrogen electrode would have a by 0.25 V lower potential due to the water influence.

The calculated equilibrium concentration of H_3O^+ is 10^{-13} M which means that only one of 4 billion protons is really coordinated by DCE and not by water.

Overall the plot can be divided into three regions:

1. For acids with pK_a values >17, the acid dissociation is dominated by a lesser extent of water protonation with higher pK_a. The ratio between [H_3O^+] and [H^+] has a constant value of $10^{8.6}$.

2. For acids with pK_a values between 8 and 17, nearly all water molecules are protonated. The overall cation concentration is dominated by that of H_3O^+,(≈10^{-4} M) and more or less independent of the pK_a. The H^+ concentration is still by orders of magnitudes lower, but increases rapidly with decreasing pK_a.

3. For acids with lower pK_a values, [H^+] becomes dominant over [H_3O^+]. E.g. for a 0.01 M acid with pK_a <6.7, the pH of the 10^{-4} M water contaminated DCE agrees within <0.1 units with that calculated for water free

9. Compatibility of the expansion. Compatibility of the expansion was tested by relative acidity measurements of acids **24** and **26**. It has been previously determined to be 0.71 and 0.73 units,[4] in this work a value of 0.69 was obtained. This indicates the comparability of experimental conditions and justifies the merging of the scales. Detailed discussion about the lower region of the scale can be found in reference [4].

10. Limitation of DCE in the high pKa end of the scale. An experimental problem becomes increasingly apparent when moving towards weak acids. The acidity scale in MeCN was composed using phosphazenes *t*-BuP$_1$(pyrr) and EtP$_2$(dma) as basic titrants[4]. The corresponding conjugate acids have pK_a values of 28.42[1] and 32.94[14] in MeCN, respectively. For studies of weaker acids than picric acid in DCE, *t*-BuP$_1$(pyrr) was first employed as basic titrant. However, it became inefficient (i.e. increasingly large titrant amounts were necessary to get detectable deprotonation) in deprotonating acids with p$K_{ip,rel}$ values above 7. Phosphazene EtP$_2$(dma) was also inefficient, but phosphazene *t*-BuP$_4$(dma) (pK_a in MeCN 42.7)[14] could be used until p$K_{ip,rel}$ value of ca 13.

For illustrating the effect of basic titrants becoming ineffective, the basic titrant consumption was plotted against pK_{ip} value in Figure S4. The consumption was calculated as molar ratio as shown in Equation S17.

$$\chi_{\text{titrant}} = \frac{n(\text{base})}{n(\text{acid}) + n(\text{TfOH})} \quad \text{(S17)}$$

In Equation S17 n(base) is the number of moles of basic titrant to titrate the acid species (n(acid) for studied acid and n(TfOH) for acidic titrant moles) into anionic form. In an ideal situation (*i.e.* the base is sufficiently strong for deprotonating the acid), this ratio should have the value of 1.

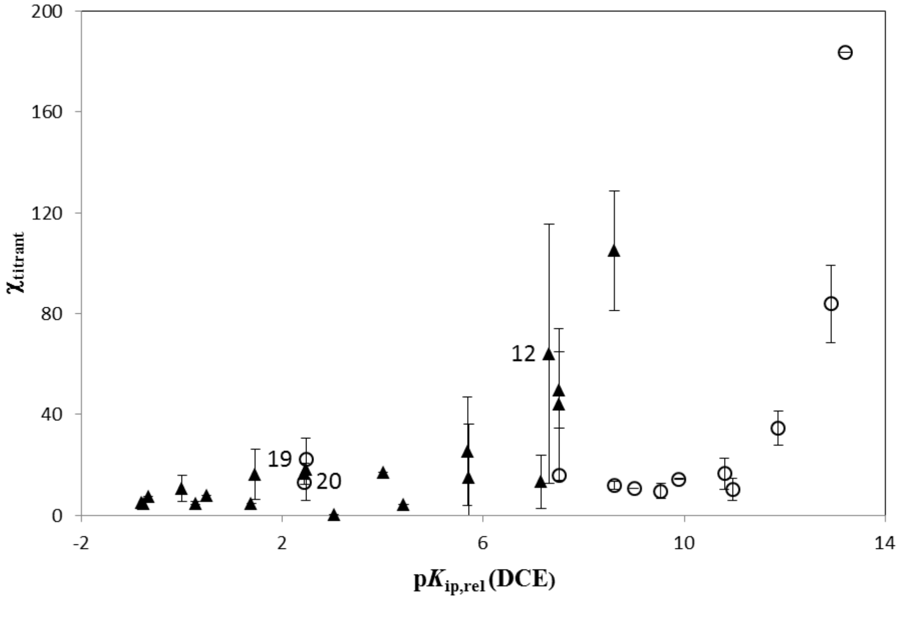

Figure S4. Basic titrant consumption dependence on p$K_{ip,rel}$ value. Triangles (▲) mark *t*-BuP$_1$(pyrr) consumption and circles (○) *t*-BuP$_4$(dma) consumption.

In Figure S4, some data points are found from individual measurements, some as averages of many measurements. For average values standard deviations are shown as error bars.

From Figure S4, it can be seen that in effective p$K_{ip,rel}$ region basic titrant consumption is 2 – 20 times more than would be expected from acid-base reaction stoichiometry. These values should be taken as values of a parameter created for describing a trend, not as absolute true values because every measurement involved some overtitration and usually very small quantities of compounds were measured, decreasing the accuracy of quantity determination. However, the $\chi_{titrant}$ values of several tens to (and beyond) 100 indicate that either (1) additional reactions (*e.g.* with the solvent or impurities in compounds) are involved when the solution is sufficiently basic for deprotonating weak acids or (2) that the measured acid has p$K_{ip,rel}$ value close to the p$K_{ip,rel}$ value of the conjugate acid of the basic titrant. High consumption of the basic titrant is additionally problematic due to deformation of spectra. In acetonitrile, *t*-BuP$_1$(pyrr) pK_a is 17.42 units higher than picric acid, but in DCE it is not possible to titrate acids with p$K_{ip,rel}$ values higher than 9 with it. Phosphazene *t*-BuP$_4$(dma) also becomes ineffective as a titrant at lower p$K_{ip,rel}$ values than would be expected from its estimated pK_a value in MeCN. This effect indicates that, not unexpectedly, neutral acids are relatively significantly weaker than cationic acids in DCE compared to MeCN.

The deviation of $\chi_{titrant}$ from 1 does not influence the experimental results as throughout the experiments only spectral data were used in calculations of ΔpK_{ip}. For acids that do not possess notable spectral changes upon deprotonation (*e.g.* acetic and benzoic acid) in the UV-Vis region an alternative experimental methodology has to be used, that brings the need for exact knowledge of the amount of titrant consumed during the titration. The inconsistency of $\chi_{titrant}$ meant that these acids, although they would have been chemically very interesting, could not be incorporated into the scale. On the other hand acids with non-existing spectral properties are included in the lower part of the acidity scale, but as can be seen from Figure S4 the $\chi_{titrant}$ gets close to 1 when p$K_{ip,rel}$ is lowered near to 0. The titrant consumption is expected to be stoichiometric when p$K_{ip,rel}$ is below 0 and this assumption is also verified by observations during the experiments in ref [4].

It was also discovered during the experiments that the solutions of all three basic titrants in DCE are stable only for limited time. This can be seen from the error bar of compound **12** in Figure S4. This acid was titrated with the same basic titrant solution fresh after making and approximately 24 hours later when titrant consumption had become 3 times larger. This refers to reaction with the solvent or its impurities or titrant decomposition. Titration of compounds **19** and **20** had similar basic titrant consumptions around 10 – 15 with both *t*-BuP$_1$(pyrr) and *t*-BuP$_4$(dma). Therefore, base/acid molar ratio parameter value of 2 – 20 can be considered regular for the experiment conducted in this work.

In order to further expand the scale, methyllithium (MeLi) was tested as basic titrant. Although it appeared to be possible to deprotonate very weak acids (with estimated p$K_{ip,rel}$ value around 15), titration spectra indicated that additional equilibria were present in the solution. This is not unexpected, based on the highly localized charge of the Li^+ ion and therefore high likelihood to form not only ion-pairs but also larger aggregates and to be selectively solvated by impurities

present in the solvent (taking into account the low solvating power of DCE). Test titration of acids **19** and **20** with MeLi yielded no quantifiable results. Relative acidity of compounds **19** and **20** was also measured using *t*-BuP_1(pyrr) and *t*-BuP_4(dma) as basic titrant. Relative acidities of 0.01 and 0.11 were determined, respectively. These results are consistent enough, considering the consistency standard deviation, to expect the relative acidities determined with either *t*-BuP_1(pyrr) or *t*-BuP_4(dma) to be directly comparable. For further expansion of the scale alternative titration technique has to be used. Probably a suitable anionic base (*i.e.* salt of some weak acid anion and large inert cation) has to be found as there are very few neutral bases, that are stronger than *t*-BuP_4(dma), at the same time easily accessible, and also exhibit low hydrogen bond donicity in the protonated form. Another possibility is to use salts of the studied acids (*e.g.* **18** and **26**) avoiding the use of basic titrant if no self-neutralization is detected. Care has to be taken in the choice of appropriate cation for the salt formation. The absence of sufficiently basic titrant also takes away the possibility to check the reversibility of the titration reaction.

11. Solvent and substituent effects on acidity. Besides MeCN and the gas phase, many acids studied in this work have also been studied in dimethyl sulfoxide (DMSO) and heptane (C7). Corresponding acidity data is given in Table S1.

Table S1. Acidity data of the acids studied in this work for different media.

	Acid	$pK_{ip,rel}$ (DCE)	$pK_{a,MeCN}$ [a]	*GA* (kcal/mol) [b]	$pK_{a,DMSO}$ [b]	$pK_{ip,rel}$ (C7) [k]
**1**	9-COOMe-fluorene	13.2	23.53		10.35 [l]	
**2**	$(4\text{-}Me\text{-}C_6F_4)_2CHCN$	12.9	22.80			4.61
**3**	$(4\text{-}Me\text{-}C_6F_4)(C_6F_5)CHCN$	11.9	21.94	316.1		3.29
**4**	9-CN-fluorene	11.0	21.36	321.4	8.3	
**5**	$(4\text{-}H\text{-}C_6F_4)(C_6F_5)CHCN$	10.8	21.11			
**6**	$(4\text{-}Cl\text{-}C_6F_4)(C_6F_5)CHCN$	9.9	20.36	311.8	7.5	1.09
**7**	$(2\text{-}C_{10}F_7)(C_6F_5)CHCN$	9.5	20.08			
**8**	9-C_6F_5-octafluorofluorene	9.0	18.88	300.6 [d]		0.00
**9**	$(2\text{-}C_{10}F_7)_2CHCN$	8.6	19.32	304.2 [g]		-0.68
**10**	$(4\text{-}CF_3\text{-}C_6F_4)(C_6F_5)CHCN$	7.5	18.14	307.5	4.9	-1.39
**11**	$(C_6F_5)CH(CN)COOEt$	7.5	17.75	313.5 [c]	4.7	
**12**	$(4\text{-}Cl\text{-}C_6F_4)CH(CN)COOEt$	7.3	17.39	312.5	4.5	
**13**	$(2\text{-}C_{10}F_7)CH(CN)COOEt$	7.1	17.50			
**14**	$(4\text{-}CF_3\text{-}C_6F_4)CH(CN)COOEt$	5.7	16.08	307.8	3.0	
**15**	$(4\text{-}NC_5F_4)(C_6F_5)CHCN$	5.7	16.40	305.7	3.3	
**16**	$(4\text{-}NC_5F_4)CH(CN)COOEt$	4.4	14.90	303.5	3.2	
**17**	$3\text{-}CF_3\text{-}C_6H_4CH(CN)_2$	4.0	14.72	307.0 [c]		
**18**	$(CF3)_5C_6CH(CN)COOEt$	3.0				
**19**	$(4\text{-}NC_5F_4)_2CHCN$	2.5	13.47	302.2	2.4	
**20**	$4\text{-}H\text{-}C_6F_4CH(CN)_2$	2.4	12.98	305.5		
**21**	$2\text{-}C_{10}F_7CH(CN)_2$	1.5	12.23	301.8		
**22**	Bromothymol blue	1.4	11.7 [f]			
**23**	Bromocresol green	0.5	11.0 [f]			
**24**	Picric acid	0.0	11.00	302.8 [c]	-1.09 [f]	
**25**	HCl	-0.4	10.30 [e]	328.1 [d]		
**26**	$2,3,4,6\text{-}(CF_3)_4\text{-}C_6HCH(CN)_2$	-0.7	10.45 [e]			

**27**	4-CF_3-$C_6F_4CH(CN)_2$	-0.8	10.19	301.5	1.7
**28**	Styphnic acid	-0.9			
**29**	4-NO_2-$C_6H_4SO_2NHTos$ [m]	-1.5	10.04	301.2 [g]	
**30**	HNO_3	-1.7		317.8 [c]	
**31**	4-NO_2-$C_6H_4SO_2NHSO_2C_6H_4$-4-Cl	-2.4	9.17	297.0 [g]	
**32**	H_2SO_4	-2.5		302.3 [c]	
**33**	$C_6(CF_3)_5CH(CN)_2$	-2.6	8.86 [e]		
**34**	(4-NO_2-C_6H_4-$SO_2)_2NH$	-3.7	8.19 [e]	291.1 [g]	
**35**	3-NO_2-4-Cl-$C_6H_3SO_2NHSO_2C_6H_4$-4-	-4.1	7.84 [e]		
**36**	(3-NO_2-4-Cl-$C_6H_3SO_2)_2NH$	-4.5	7.47 [e]		
**37**	HBr	-4.9	5.5 [e]	318.3 [c]	
**38**	4-NO_2-$C_6H_4SO_2CH(CN)_2$	-5.1	6.06 [e]		
**39**	2,4,6-$(SO_2F)_3$-Phenol	-5.9	5.25 [e]		
**40**	2,4,6-Tf_3-Phenol [n]	-6.4	4.48 [e]		
**41**	$CH(CN)_3$	-6.5		287.6 [i]	
**42**	4-Cl-C_6H_4SO(=NTf)NHTos	-6.8	4.81 [e]		
**43**	NH_2-TCNP [o]	-6.8			
**44**	2,3,5-tricyanocyclopentadiene	-7.0	3.68 [e]		
**45**	Pentacyanophenol	-7.6			
**46**	4-Cl-C_6H_4SO(=NTf)$NHSO_2C_6H_4$-4-Cl	-7.6	4.03 [e]		
**47**	HI	-7.7	2.8 [e]	309.2 [c]	
**48**	4-NO_2-$C_6H_4SO_2NHTf$	-7.8	4.08 [e]		
**49**	Me-TCNP	-8.6			
**50**	3,4-$(MeO)_2$-C_6H_3-TCNP	-8.7			
**51**	4-MeO-C_6H_4-TCNP	-8.7			
**52**	$C(CN)_2$=C(CN)OH	-8.8	4.39 [e]	283.2 [j]	
**53**	4-Cl-C_6H_4SO(=NTf)$NHSO_2C_6H_4$-NO_2	-8.8	3.33 [e]		
**54**	2,4-$(NO_2)_2$-$C_6H_3SO_2OH$	-8.9	3.99 [e]		
**55**	$C_6F_5CH(Tf)_2$	-9.0			
**56**	$HB(CN)(CF_3)_3$	-9.3			
**57**	Ph-TCNP	-9.4			
**58**	HBF_4	-10.3		288.5 [i]	
**59**	FSO_2OH	-10.5		295.4 [i]	
**60**	3-CF_3-C_6H_4-TCNP	-10.5			
**61**	H-TCNP	-10.7			
**62**	[C_6H_5SO(=NTf)]$_2$NH	-11.1			
**63**	[(C_2F_5)2PO]$_2$NH	-11.3			
**64**	2,4,6-$(NO_2)_3$-$C_6H_2SO_2OH$	-11.3			
**65**	[$C(CN)_2$=C(CN)]$_2CH_2$	-11.4			
**66**	TfOH	-11.4		293.3 [i]	
**67**	C_6H_5SO(=NTf)NHTf	-11.5			
**68**	$TfCH(CN)_2$	-11.6		282.2 [i]	
**69**	Br-TCNP	-11.8			
**70**	[$C(CN)_2$=C(CN)]$_2$NH	-11.8			
**71**	3,5-$(CF_3)_5$-C_6H_3-TCNP	-11.8			
**72**	Tf_2NH	-11.9		286.5 [g]	
**73**	4-Cl-C_6H_4SO(=NTf)NHTf	-12.1			
**74**	Cl-TCNP	-12.1			
**75**	$(C_3F_7SO_2)_2NH$	-12.1		281.1 [h]	

**76**	$(C_4F_9SO_2)_2NH$	-12.2	278.7 [h]
**77**	CN-CH_2-TCNP	-12.3	
**78**	$(C_2F_5SO_2)_2NH$	-12.3	283.7 [g]
**79**	CF_3-TCNP	-12.7	
**80**	$HClO_4$	-13.0	295.0 [i]
**81**	$CF_2(CF_2SO_2)_2NH$	-13.1	284.2 [g]
**82**	4-NO_2-C_6H_4SO(=NTf)NHTf	-13.1	
**83**	$HB(CN)_4$	-13.3	
**84**	$(FSO_2)_3CH$	-13.6	292.2 [i]
**85**	Tf_2CH(CN)	-14.9	
**86**	2,3,4,5-tetracyanocyclopentadiene	-15.1	
**87**	CN-TCNP	-15.6	267.2 [i]

[a] From ref [7] if not specified otherwise; [b] From ref [15] if not specified otherwise; [c] From ref [16]; [d] From ref [17]; [e] From ref [4]; [f] From ref [18]; [g] From ref [19]; [h] From ref [20]; [i] From ref [21]; [j] From ref [22]; [k] From ref [1]; [l] From ref [23]; [m] Tos represents 4-Me-$C_6H_4SO_2$- group; [n] Tf represents CF_3SO_2- group; [o] X-TCNP represents 2-X-1,1,3,3-tetracyanopropene.

Thermodynamic relations between different acids are given in S-BFHC 3:

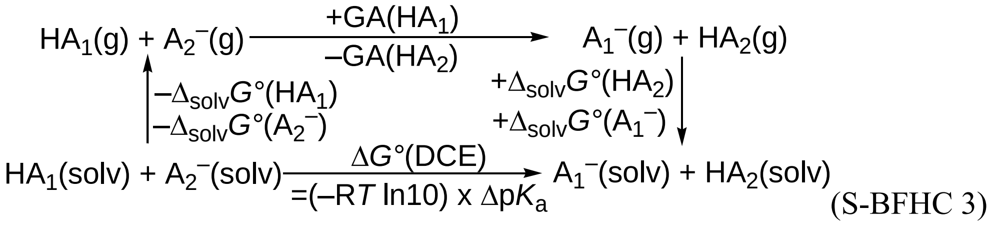

(S-BFHC 3)

Correlation of $pK_{ip,rel}$ values in DCE and pK_a values in MeCN is given in Figure S5.

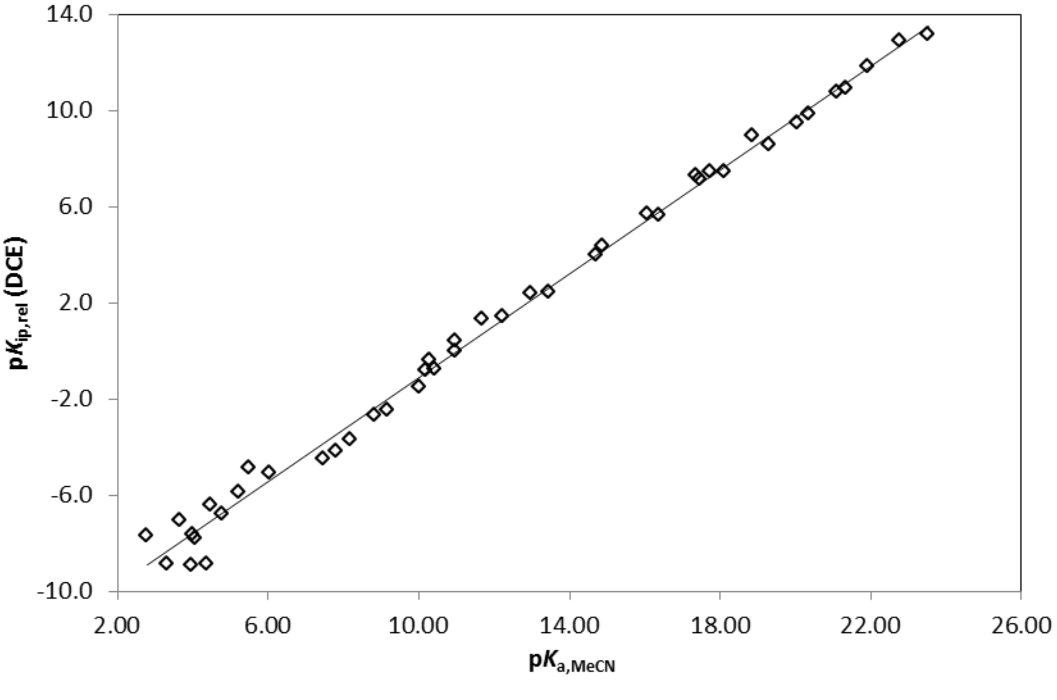

Figure S5. Correlation of the acidities of studied acids in DCE and MeCN medium. Solid line (——) corresponds to the overall correlation.

The correlation of acidities in DCE and MeCN:

$$pK_{ip,rel}(DCE) = 1.08\ pK_{a,MeCN} - 11.9 \quad (S18)$$

s(slope) = 0.01; s(intercept) = 0.2; n = 44; R^2 = 0.994; S = 0.6

$$pK_{a,rel}\ (DCE) = 1.08\ pK_{a,MeCN} - 12.0 \quad (S19)$$

s(slope) = 0.02; s(intercept) = 0.2; n = 44; R^2 = 0.992; S = 0.6

The small decline in the quality of correlation in Eq S19 is largely due to increased deviation of hydrogen halide acidities as a result of correction for ion-pairing. In both cases the overall correlation is good. The upper part of the scale is composed almost exclusively of CH acids, which give large anions with delocalized charge upon ionization. Neither of the solvents give specific interactions with these acids or their anions and the effect of ion pairing with the inert cation t-BuP$_1$(pyrr)H$^+$ is similar between the acid anions, resulting in especially good correlation in that part of the scale. In the lower part of the scale ($pK_{ip,rel}$(DCE) < 0) there are a number of acids having small anions or anions with localized charge. These anions (as well as the acids) are more prone to participating in specific interactions and thus the correlation in the lower part of the scale is satisfactory. The slope of the correlation describes the difference of differentiating ability of the solvents.

Correlation of $pK_{ip,rel}$ values in DCE and GA values is given in Figure S6.

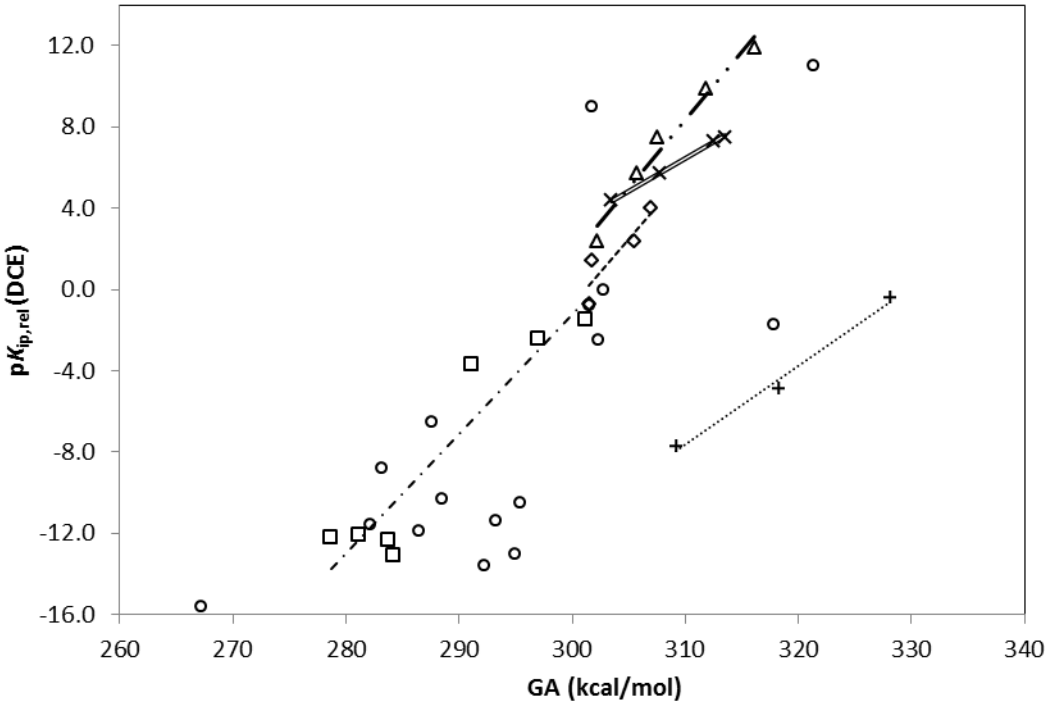

Figure S6. Correlation between the acidities of the studied acids in DCE and the gas phase. Dotted line (··) corresponds to hydrogen halides (+), dashed line (- - -) to arylmalonnitriles (◊), dash-dot line (-·-) to sulfonimides (□), dot-dot-dash line (-··-)to arylacetonitriles (Δ) and double line (══) to ethyl aryl cyanoacetates (x).

Correlation parameters for different acid families are given in Table S2.

Table S2. p$K_{ip,rel}$(DCE) versus *GA* (kcal mol^{-1}) correlations of different acid families.

Acid family	slope	intercept	*s*(slope)	*s*(intercept)	*n*	R^2	*S*
Diarylacetonitriles	0.67	-199	0.07	21	5	0.969	0.74
Arylmalonnitriles	0.65	-197	0.2	65	4	0.825	1.01
Ethyl aryl cyanoacetates	0.32	-91	0.01	3	4	0.999	0.07
Sulfonimides	0.59	-178	0.1	28	7	0.881	2.01
Hydrogen halides	0.39	-128	0.04	14	3	0.987	0.58

For correlations given in Table S2, lower slope value refers to acidities being more strongly dependent on solvation. Out of the studied families, the acidities of ethyl aryl cyanoacetates are the most dependent on solvation. This can be explained by the fact that in the anions of these acids a considerable share of the negative charge is localized on the carbonyl oxygen. Thus, solvent has a larger role in stabilization of the negative charge. Acidity of hydrogen halides is similarly very dependent on solvation due to small anion size and thus high charge localization. The differentiating ability of DCE with respect to these two acid families is less than half of that in the gas phase. Acidities of diarylacetonitriles and arylmalonnitriles are less dependent on solvent due to more effective charge delocalization in anions. The differentiating ability of DCE for the acids from these families is around 70% of that of the gas phase.

Ion-pair acidities can be compared between DCE and heptane. The obtained correlation is given in Figure S7.

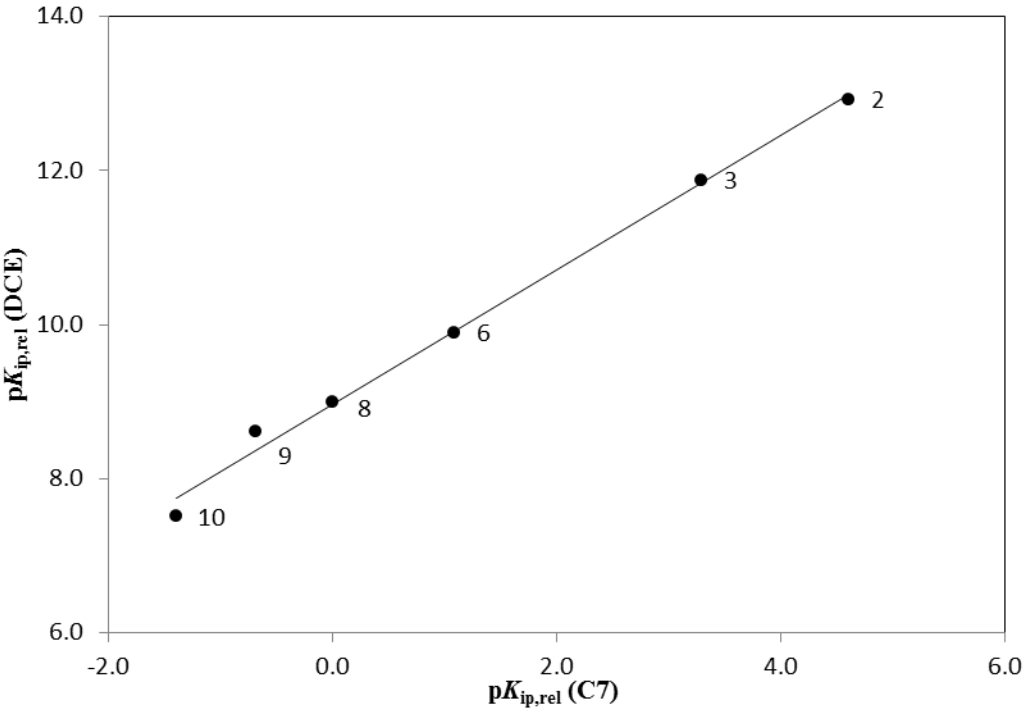

Figure S7. Correlation of the acidities of studied acids in DCE and heptane medium. Narrow line (——) corresponds to the overall correlation.

The correlation line in Figure S7 is described by Equation 20.

$$pK_{ip,rel}(DCE) = 0.87\ pK_{ip,rel}(C7) + 8.96 \quad (20)$$

s(slope) = 0.03; s(intercept) = 0.08; n = 6; R^2 = 0.994; S = 0.18

It can be evaluated that DCE is more than 10% less differentiating solvent than heptane. Due to small data set it cannot be determined more accurately.

There is significant amount of acidity data in DMSO, especially of weak acids.[23] The $pK_{ip,rel}$ values of studied acids are plotted against DMSO pK_a values in Figure S8.

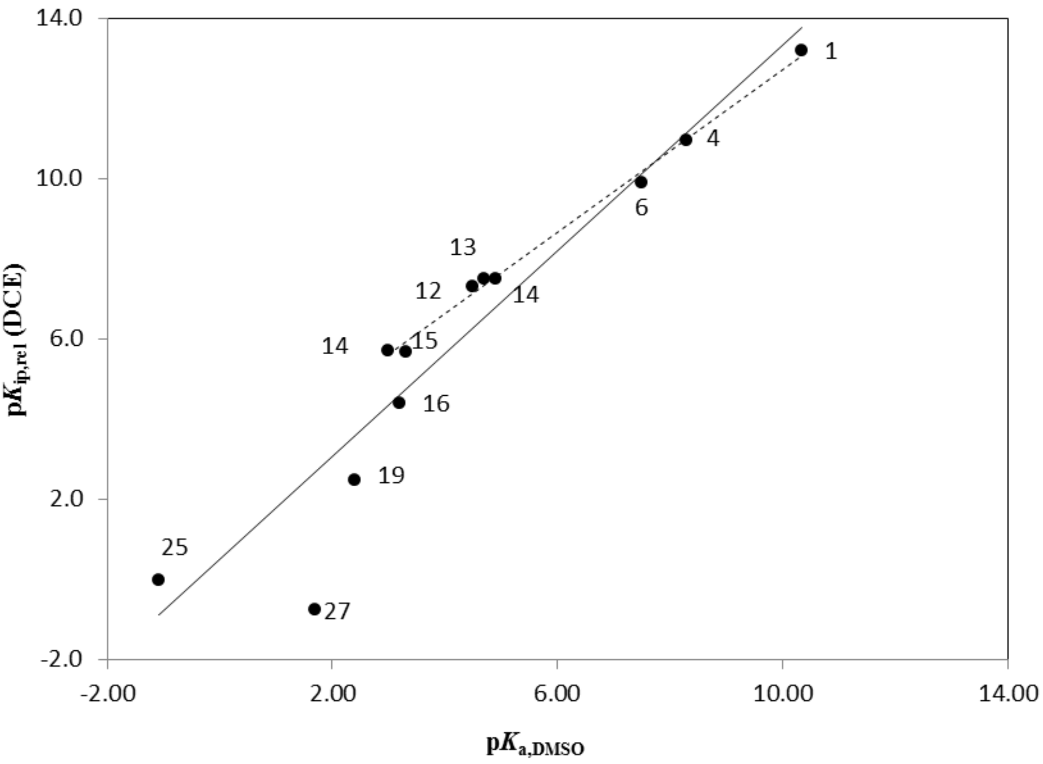

Figure S8. Correlation of the acidities of studied acids in DCE and DMSO medium. Narrow line (—) corresponds to the overall correlation. After removing acids 16, 19, 25 and 27 a better correlation (-----) is obtained.

Overall correlation of acidities in DCE and DMSO is described by Equation S21.

$$\mathrm{p}K_{\mathrm{ip,rel}}(\mathrm{DCE}) = 1.28\ \mathrm{p}K_{\mathrm{a,DMSO}} + 0.5 \qquad \text{(S21)}$$

s(slope) = 0.14; s(intercept) = 0.7; n = 12; R^2 = 0.901; S = 1.40

Acidities in DCE appear to correlate not as well with DMSO acidities as with MeCN and heptane. This is probably due to higher basicity of DMSO that causes overestimation of $\mathrm{p}K_{\mathrm{a,DMSO}}$ values of stronger acids. It has been shown that $\mathrm{p}K_{\mathrm{a}}$ values less than 2 units are usually overestimated with potentiometric titration[15] (which has been widely employed in DMSO). This is likely the reason for deviating values of **16** and **19**. Acidities of **24** and **27** have not been obtained experimentally but with indirect methods.[15,18] Leaving out those four mentioned compounds, a better correlation is received:

$$\mathrm{p}K_{\mathrm{ip,rel}}(\mathrm{DCE}) = 1.02\ \mathrm{p}K_{\mathrm{a,DMSO}} + 2.6 \qquad \text{(S22)}$$

s(slope) = 0.03; s(intercept) = 0.2; n = 8; R^2 = 0.996; S = 0.19

Equation S22 implies that DCE has similar differentiating ability to DMSO.

In the present work, acids with insignificant hydrogen bond donicity, that have anions with extensively delocalized charge, were studied. Relative acidities of these acids are relatively

independent of solvation and good correlations are expected. These correlations make it possible for different acidity scales to complement each other.

12. Experimental. Relative acidities expressed as ΔpK_{ip} values were measured using a spectrophotometric titration method and calculation methods essentially identical to that used previously[4] (in previous work[4] these values are denoted as ΔpK_a). The experiments were conducted in a glovebox under argon atmosphere.

The expansion of the acidity scale was composed using mostly acids with CH acidity center, devoid of specific solvation of anionic and neutral forms and having very different UV-Vis spectra of anion and neutral, enabling accurate spectrophotometric measurements. Most of the acids used for composing the scale are the same as the ones used in ref [4]. Synthesis of compound **18** is presented below. Compound **22** is from Reakhim (grade "pure for analysis") and **23** from Sigma-Aldrich (95%) and both were used as received. Compound **28** is a gift from Prof. Tullio Ilomets.

Protonation-deprotonation reversibility was verified with all the acids. In the case of fluorenes slow decomposition was observed in the fully ionized state. The effect of this was minimized by conducting the titration more rapidly.

The purity of acids was evaluated from the UV-Vis spectra. Most acids displayed sharp isosbestic points, which was used as indication of sufficient purity for pK_a measurements. It is possible that 3-trifluoromethylphenylmalononitrile (**17**) contained some impurities (as indicated by the spectra), but as relative acidities measured against **17** were consistent, it was included in the scale.

Two acids (**18** and **26**) were used as Et_4N^+ salts. Considering the fact that ion-pair acidities were measured, there may had been an equilibrium between Et_4N^+ ion and protonated titrant ion and thus the $pK_{ip,rel}$ values determined for those acids may be intrinsic for the mixture of two corresponding ion-pairs. However, this is not expected to substantially influence the results because both cations are inert and do not give specific interactions.

Environment temperature varied between 22 – 25 °C throughout the experiments. The solution temperature was also measured at irregular intervals and was usually 0.5 – 1 °C higher than the temperature of the environment, *i.e.* varied between 23 – 26 °C. This means that the temperature of the measurements can be conservatively estimated as (25 ± 3) °C. Water content of the used solvent was always less than 5 ppm, often less than 2 ppm (determined by coulometric KF titration).

The low water content was achieved by drying commercial DCE (water content <0.01%) with molecular sieves for at least 24 hours before the experiment. Because of the low polarity and low hydrogen bond donor and acceptor properties of DCE traces of water in this solvent have stronger influence on acid-base processes than in e.g. DMSO or acetonitrile.[4] Most of that influence is expected to come from solvation of the proton and cancels out in the case of the relative measurement approach used in this work.

The origin of compounds and comments about the experiments in the lower part of the scale (pK_{ip}<0) can be found in ref [4].

13. Synthesis of compound 18. A suspension of NaH (3 mmol, 0.072 g) in dry THF (1 mL) was consequently treated with ethyl 2-cyanoacetate (1.42 mmol, 0.16 g) dissolved in dry THF (4ml) and then with the solution of $C_6(CF_3)_5Cl$[24] (1.33 mmol, 0.6 g) dissolved in dry THF (6 mL) at -78 °C under argon environment. The mixture turned yellow at the beginning and after warming up to room temperature the mixture turned dark red. ^{19}F NMR spectra were recorded after two hours and after five hours, both spectra were almost identical showing that a lot of $C_6(CF_3)_5Cl$ has not reacted, but some amount of new compound appeared as well.

The solution was then filtrated to remove NaCl and excessive NaH. From filtrate all the volatiles were very carefully removed *in vacuo*. The residue was dissolved in water (10 ml), filtered to remove remaining $C_6(CF_3)_5Cl$, and then solid Et_4NBr (1.48 mmol, 0.31 g) was added to the solution. A dark oily substance formed, which turned solid after cooling the solution to 0 °C. Removing of water via filtration followed by recrystallization from EtOH/water mixture (1:7) afforeded the target product. Yield: 18% (0.23 mmol, 0.15 g).

For UV-Vis measurements and for the analysis the compound was additionally purified chromatographically using Al_2O_3 as stationary phase and the 1:1 mixture of AcOEt:MeCN as eluent. Some impurities remained to the top of the column, some eluted very fast. The dark red fraction was collected and solvents removed *in vacuo*.

^{13}C{^{1}H} NMR (176 MHz, Acetonitrile-d_3) δ = 166.63 (s, O-*C*=O), 149.52 (s, *i*-C), 130.95 (qm, $^{2}J_{C\text{-}F}$ = 35.5 Hz,*o*-C_{Ar}), 123.47 (q, $^{1}J_{C\text{-}F}$ = 275.5 Hz, *m*-CF_3), 124.56 (q, $^{1}J_{C\text{-}F}$ = 272.9 Hz, *p*-CF_3), 123.16 (q, $^{1}J_{C\text{-}F}$ = 273.4 Hz, *o*-CF_3), 119.38 (qm, $^{2}J_{C\text{-}F}$ = 32.9 Hz, *m*-C_{Ar}), 115.67 (qm, $^{2}J_{C\text{-}F}$ = 34.6 Hz, *p*-C_{Ar}), 72.65 (s, *C*N), 60.00 (s,O*C*H_2CH_3), 53.08 (s,N*C*H_2CH_3), 14.84 (s, OCH_2*C*H_3), 7.65 (s, NCH_2*C*H_3)

^{1}H NMR (700 MHz, Acetonitrile-d_3) δ = 3.99 (q, $^{3}J_{HH}$ = 7.1 Hz, 2H, OCH_2CH_3), 3.16 (q, $^{3}J_{HH}$ = 7.3 Hz, 8H, NCH_2CH_3), 1.20 (qt, $^{3}J_{HH}$ = 7.3 Hz, $^{3}J_{H\text{-}^{14}N}$ = 1.9 Hz, 12H, NCH_2CH_3), 1.13 (t, $^{3}J_{HH}$ = 7.1 Hz, 3H, OCH_2CH_3).

^{19}F NMR (659 MHz, Acetonitrile-d_3, C_6F_6 = -162.59 ppm) δ = -47.39 (hept, $^{5}J_{FF}$ = 15.8 Hz, 3F, *p*-CF_3), -51.79 (q, $^{5}J_{FF}$ = 16.6 Hz, 6F, *o*-CF_3), -51.27 (hept, $^{5}J_{FF}$ = 16.3 Hz, 6F, *m*-CF_3).

HRMS-ESI *m/z*: M^- calcd for $C_{16}H_5F_{15}NO_2^-$, 528.00862; found, 528.00864; Et_4N^+ calcd for $C_8H_{20}N^+$, 130.15903 found, 130.15903.

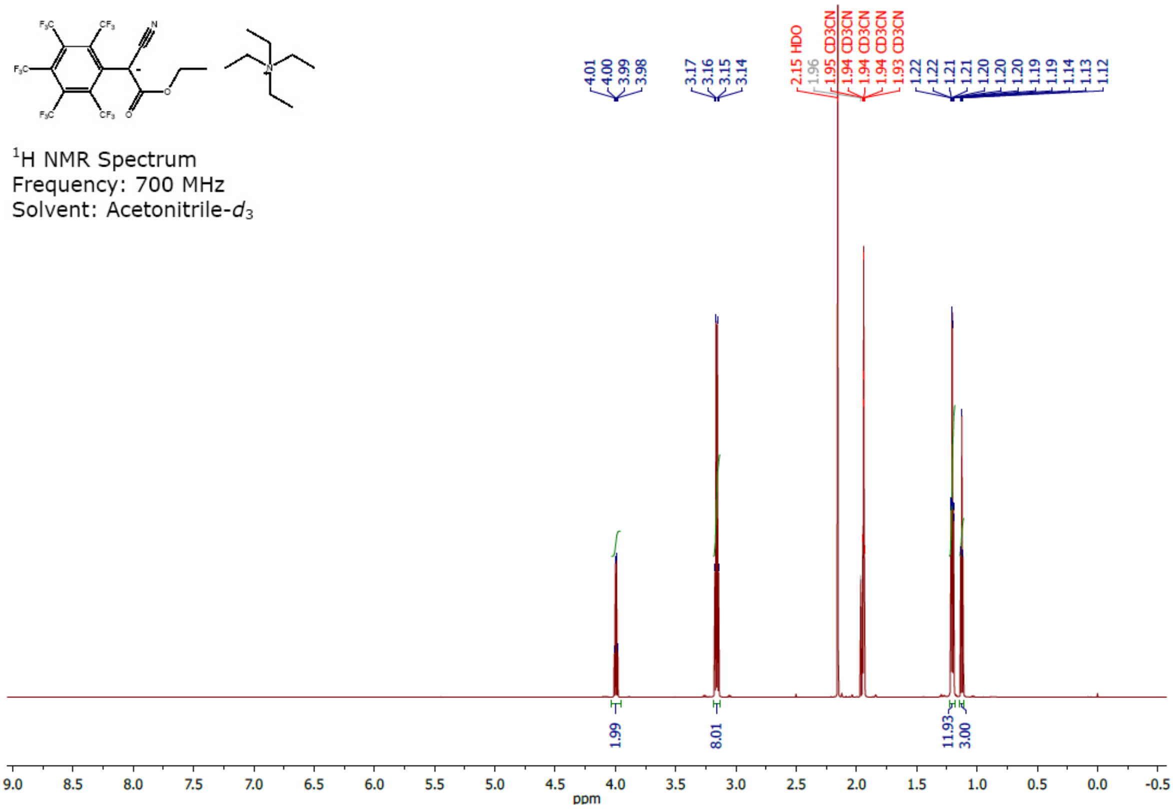

^{1}H NMR Spectrum
Frequency: 700 MHz
Solvent: Acetonitrile-d_3

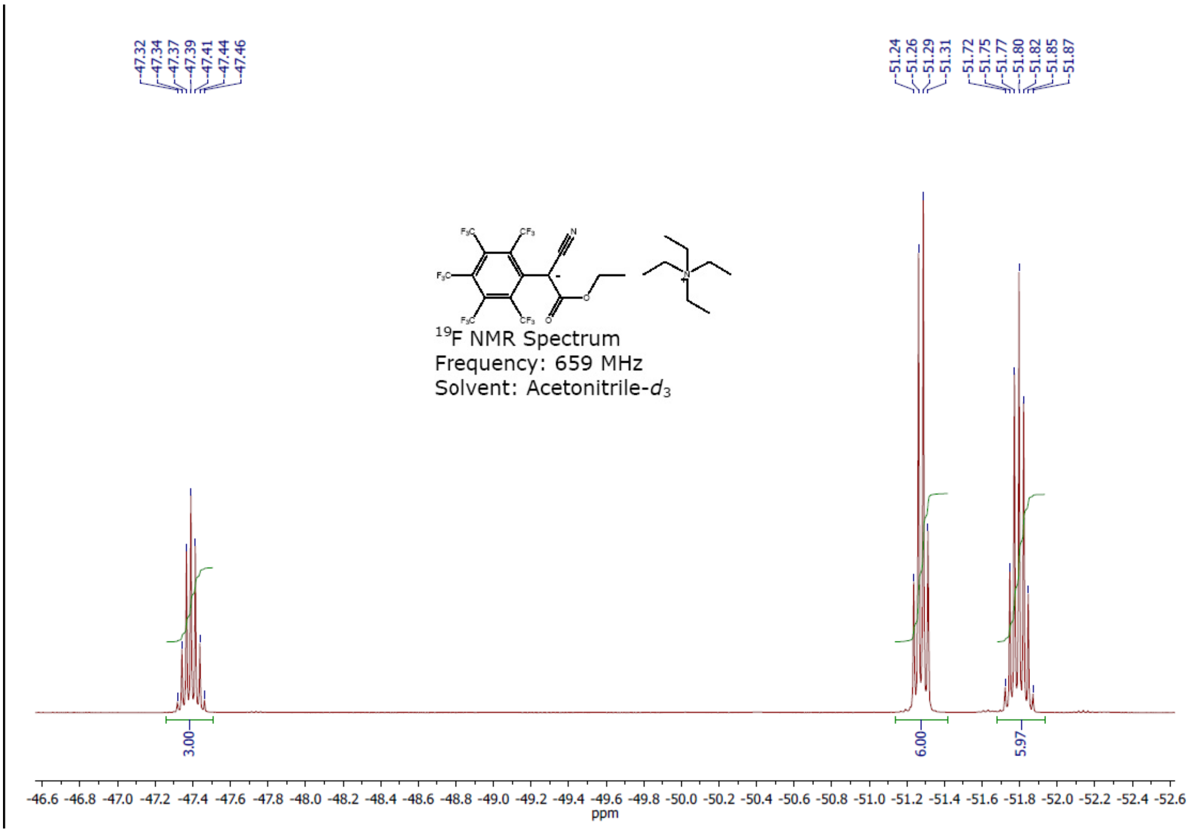

^{19}F NMR Spectrum
Frequency: 659 MHz
Solvent: Acetonitrile-d_3

14. Details on the quantum chemical calculations.

Note: Values in the BFHCs are $\Delta G°$ in kJ mol^{-1}, unless otherwise indicated.

A. SMD calculations on pK_a(H_3O^+, DCE). The general Methodology is described in Ref. 25.

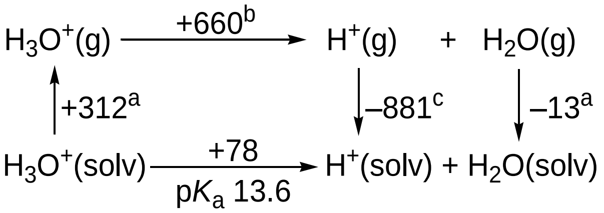

(S-BFHC 4)

[a] This work, see Table S3 below; [b] see Ref. 26; [c] see Ref. 25

Table S3: B3LYP/6-311+G** SCF energies

	SCF(gas) [H]	SCF(DCE) [H]	$\Delta_{solv}G°$ [kJ mol^{-1}]
H_3O^+	-76.7310716	-76.852999	-312.2
H_2O	-76.4584627	-76.466541	-13.3

B. COSMO-RS calculations

COSMO-RS[27] input files (.cosmo and .energy) were created with the COSMO[28] module of the Turbomole[29] program system according to the “BP_TZVPD_FINE_C30_1501.ctd” formalism. The COSMOTherm[30] program was used to calculate COSMO-RS Gibbs solvation energies.

COSMO-RS $\Delta_{solv}G°$ were calculated from “single ion vapour pressures over the mixture” (see “Log10(partial pressure [kPa])” in Cosmotherm output), denoted here as $p_{vap}°$ with the relation:

$\Delta_{solv}G°(X) = RT \ln((p_{vap}°(X) \cdot x°(X))/1 \text{ bar})$ with $x°(X) = 1$ for the solvent DCE (pure liquid standard state) and 0.0792 (mole fraction of an ideal one-molar solution as standard state) for solutes.

Thermodynamics of the proton is given in S-BFHC 5 (solvation thermodynamics from Table S4):

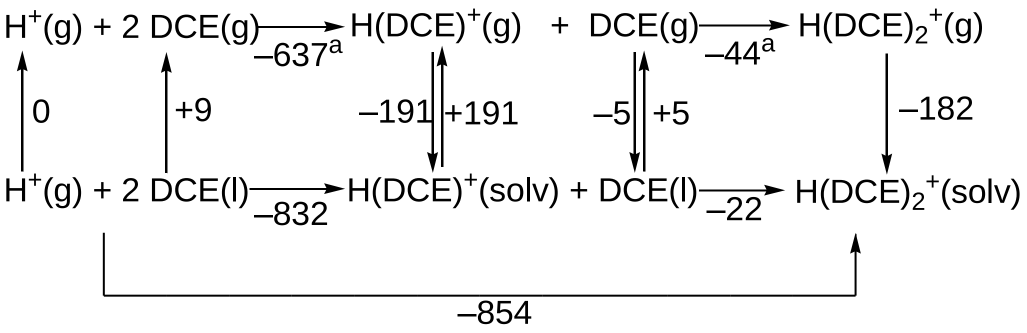

(S-BFHC 5)

[a] G3(mod) level of theory, see G3[31].

S-BFHC 6 shows the protolysis thermodynamics of HNTf2 in DCE (solvation thermodynamics from Table S4):

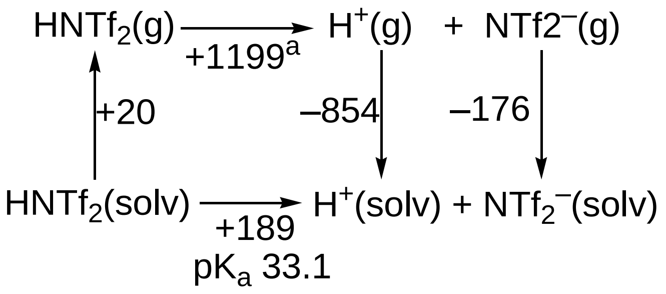

(S-BFHC 6)

[a] Exp. value, see Ref. [19]

Table S4. COSMOTherm results.

Compound	lgp[kPa]	x°	p(x°) [bar]	$\Delta_{solv}G°$ [kJ mol^{-1}]
DCE in DCE	1.20937	1	$1.619\cdot10^{-1}$	–4.5
HNTf2 in DCE	–0.47017	0.0792	$2.682\cdot10^{-4}$	–20.4
NTf2– in DCE	–27.76178	0.0792	$1.370\cdot10^{-31}$	–176.2
HDCE+ in DCE	–31.72436	0.0792	$1.494\cdot10^{-35}$	–198.8
H(DCE)2+ in DCE	–28.8165	0.0792	$1.208\cdot10^{-32}$	–182.2

Optimized BP86-D3/def-TZVP cartesian coordinates (Å)

DCE, conformer 1

C	-0.479965910	-0.585866403	0.000000001
C	0.479965910	0.585866403	-0.000000001
Cl	-2.190312865	0.059588537	-0.000000000
Cl	2.190312865	-0.059588537	0.000000000
H	-0.365727442	-1.203930373	0.896865251
H	-0.365727442	-1.203930373	-0.896865252
H	0.365727442	1.203930373	-0.896865251

H	0.365727442	1.203930373	0.896865252

DCE, conformer 2

C	-0.754521861	-0.048666344	-0.131123435
C	0.754521861	0.048666344	-0.131123435
H	-1.084899630	-0.537089339	-1.058231573
H	-1.221869279	0.939280451	-0.050752957
H	1.084899630	0.537089339	-1.058231573
H	1.221869278	-0.939280451	-0.050752957
Cl	-1.398428176	-1.056846430	1.241358041
Cl	1.398428176	1.056846430	1.241358041

$HNTf_2$, conformer 1

S	-1.528758289	-0.345451355	0.009067960
N	0.000000181	-0.000028107	0.738084127
S	1.528748573	0.345454030	0.009060340
O	-2.323151990	-0.974076550	1.082110735
O	-1.304079693	-0.911954470	-1.328161014
O	2.323152733	0.974103874	1.082110322
O	1.304077922	0.911976281	-1.328158934
C	-2.228621326	1.448989383	-0.220296658
F	-2.225745558	2.065009591	0.972417588
F	-3.480380414	1.347117076	-0.680823428
F	-1.469330225	2.124432855	-1.084209922
C	2.228636495	-1.449003213	-0.220303610
F	1.469300214	-2.124440079	-1.084200218
F	3.480396764	-1.347146028	-0.680835162
F	2.225743002	-2.064975336	0.972436830
H	0.000011609	-0.000007951	1.761701044

$HNTf_2$, conformer 2

S	-1.274264750	-1.148911120	-0.110408674
N	0.152788537	-0.623101543	0.736908686
S	1.800322452	-0.611291900	0.162757659
O	-1.924870808	-2.183443159	0.716051547
O	-0.959510411	-1.272537301	-1.541048802
O	1.995342154	-1.555268363	-0.946445163
O	2.596373299	-0.597543022	1.406684344
C	-2.319667533	0.467686179	0.129114011
F	-2.420767174	0.728937358	1.442550134
F	-3.533451900	0.257044410	-0.390668841
F	-1.723641739	1.490931785	-0.492636136
C	1.855625734	1.174502375	-0.587025109
F	1.542962747	2.065676777	0.361225240
F	3.100944185	1.388411835	-1.026440514
F	0.987351010	1.256632946	-1.599966609
H	0.124464196	-0.837727255	1.739348226

NTf_2^-, conformer 1

S	-1.407638806	-0.325661326	0.130642010
N	0.000002794	-0.000003340	0.929413494
S	1.407639879	0.325666089	0.130637776
O	-2.318831667	-0.992069269	1.101545951
O	-1.312709405	-0.840712661	-1.264266165
O	2.318822961	0.992060761	1.101537029
O	1.312698895	0.840721523	-1.264280316
C	-2.191656168	1.433783181	-0.069754207
F	-2.427387738	2.001864807	1.136098222
F	-3.378835312	1.331701140	-0.723454920
F	-1.389834688	2.256232841	-0.777170714
C	2.191659772	-1.433785365	-0.069753988
F	1.389845907	-2.256231034	-0.777176065
F	3.378843958	-1.331701283	-0.723445768
F	2.427379617	-2.001866063	1.136099679

NTf_2^-, conformer 2

S	-1.218075887	-1.291666298	0.488286115
N	0.257714052	-0.726504587	0.930231092
S	1.523642802	-0.603619066	-0.123686677
O	-1.997846867	-1.481905579	1.742085341
O	-1.236600354	-2.346678610	-0.563005737
O	1.238987994	-0.737290699	-1.580869226
O	2.730510762	-1.264180403	0.448743140
C	-2.117738410	0.210378834	-0.344491080
F	-2.152566088	1.276135398	0.490525276
F	-3.400566081	-0.133859276	-0.635554805
F	-1.514307604	0.586969514	-1.490196346
C	1.894525571	1.277827828	0.123382662
F	2.222343066	1.560458805	1.403594206
F	2.940250917	1.637414339	-0.666530454
F	0.829726128	2.036519800	-0.222513507

$H(DCE)^+$

C	0.685676468	-0.323846023	-1.155255185
C	-0.685676467	0.323846024	-1.155255184
Cl	1.503622557	0.074769531	0.467018894
Cl	-1.503622557	-0.074769531	0.467018894
H	1.325592777	0.098440111	-1.939483293
H	0.659495169	-1.417220211	-1.202671744
H	-1.325592777	-0.098440111	-1.939483294
H	-0.659495169	1.417220210	-1.202671744
H	-0.000000000	0.000000000	0.969625490

$H(DCE)^+$

Cl	1.385997720	-0.768809219	-0.081065002

H	-0.773300460	3.254336658	1.433339701
H	0.773299840	-3.254336750	1.433338916
C	0.844283542	-2.495664260	-0.602911488
H	-0.016412194	-4.260789120	0.181375780
H	-0.203782938	2.335011055	-1.476217017
H	-0.000000066	0.000000008	-0.047155253
Cl	-1.385997630	0.768809203	-0.081065105
H	0.016412073	4.260788680	0.181375979
H	0.203783865	-2.335011250	-1.476215949
C	-0.167414532	3.230591171	0.520650233
Cl	1.443118502	2.511106932	0.940659418
C	-0.844285495	2.495665127	-0.602910006
C	0.167415443	-3.230590925	0.520651808
H	1.793364818	-2.964037978	-0.892255758
H	-1.793363968	2.964037547	-0.892255595
Cl	-1.443118521	-2.511106877	0.940659339

References

1 I. Leito, I. Kaljurand, T. Rodima, A. Kütt, A. Pihl, E.-I. Rõõm, L. Sooväli, V. Mäemets, V. Pihl and I. A. Koppel, *Proc. Est. Acad. Sci. Chem.*, 2005, **54**, 94.

2 K. Abdur-Rashid, T. P. Fong, B. Greaves, D. G. Gusev, J. G. Hinman, S. E. Landau, A. J. Lough and R. H. Morris, *J. Am. Chem. Soc.*, 2000, **122**, 9155.

3 I. Kaljurand, T. Rodima, A. Pihl, V. Maemets, I. Leito, I. A. Koppel and M. Mishima, *J. Org. Chem.*, 2003, **68**, 9988.

4 A. Kütt, T. Rodima, J. Saame, E. Raamat, V. Maemets, I. Kaljurand, I. A. Koppel, R. Y. Garlyauskayte, Y. L. Yagupolskii, L. M. Yagupolskii, E. Bernhardt, H. Willner and I. Leito, *J. Org. Chem.*, 2011, **76**, 391.

5 Spartan ´08 Wavefunction Inc., Irvine, CA, USA.

6 L. P. Hammett and A. J. Deyrup, *J. Am. Chem. Soc.*, 1932, **54**, 2721.

7 A. Kütt, I. Leito, I. Kaljurand, L. Soovali, V. M. Vlasov, L. M. Yagupolskii and I. A. Koppel, *J. Org. Chem.*, 2006, **71**, 2829.

8 I. Leito, T. Rodima, I. A. Koppel, R. Schwesinger and V. M. Vlasov, *J. Org. Chem.*, 1997, **62**, 8479.

9 D. R. Lide, ed., *CRC handbook of chemistry and physics. A ready-reference book of chemical and physical data*, CRC Taylor & Francis, Boca Raton, Fla., 87th edn., 2006.

10 J. D. D. Martin and J. W. Hepburn, *J. Chem. Phys.*, 1998, **109**, 8139.

11 P. G. T. Fogg, W. Gerrard and H. L. Clever, *IUPAC Solubility Data Series*, 240.

12 Y. Marcus, M. J. Kamlet and R. W. Taft, *J. Phys. Chem.*, 1988, **92**, 3613.

13 P. Debye and E. Hückel, *Phys. Z.*, 1923, 185.

14 R. Schwesinger, H. Schlemper, C. Hasenfratz, J. Willaredt, T. Dambacher, T. Breuer, C. Ottaway, M. Fletschinger, J. Boele, H. Fritz, D. Putzas, H. W. Rotter, F. G. Bordwell, A. V. Satish, G.-Z. Ji, E.-M. Peters, K. Peters, H. G. von Schnering and L. Walz, *Liebigs Ann./Recl.*, 1996, 1055.

15 I. A. Koppel, J. Koppel, V. Pihl, I. Leito, M. Mishima, V. M. Vlasov, L. M. Yagupolskii and R. W. Taft, *J. Chem. Soc., Perkin Trans. 2*, 2000, 1125.

16 I. A. Koppel, R. W. Taft, F. Anvia, S.-Z. Zhu, L.-Q. Hu, K.-S. Sung, D. D. DesMarteau, L. M. Yagupolskii and Y. L. Yagupolskii, *J. Am. Chem. Soc.*, 1994, **116**, 3047.

17 E. Raamat, K. Kaupmees, G. Ovsjannikov, A. Trummal, A. Kütt, J. Saame, I. Koppel, I. Kaljurand, L. Lipping, T. Rodima, V. Pihl, I. A. Koppel and I. Leito, *J. Phys. Org. Chem.*, 2013, **26**, 162.

18 K. Izutsu, *Acid-base dissociation constants in dipolar aprotic solvents*, Blackwell, Oxford, 1990, vol. 35.

19 I. Leito, E. Raamat, A. Kutt, J. Saame, K. Kipper, I. A. Koppel, I. Koppel, M. Zhang, M. Mishima, L. M. Yagupolskii, R. Y. Garlyauskayte and A. A. Filatov, *J. Phys. Chem. A*, 2009, **113**, 8421.

20 M. Zhang, T. Sonoda, M. Mishima, T. Honda, I. Leito, I. A. Koppel, W. Bonrath and T. Netscher, *J. Phys. Org. Chem.*, 2014, **27**, 676.

21 I. A. Koppel, P. Burk, I. Koppel, I. Leito, T. Sonoda and M. Mishima, *J. Am. Chem. Soc.*, 2000, **122**, 5114.

22 I. A. Koppel, P. Burk, I. Koppel and I. Leito, *J. Am. Chem. Soc.*, 2002, **124**, 5594.

23 F. G. Bordwell, *Acc. Chem. Res.*, 1988, **21**, 456.

24 a) A. Kütt, F. Werner, I. Kaljurand, I. Leito and I. A. Koppel, *ChemPlusChem*, 2013, **78**, 932; b) A. Kütt, V. Movchun, T. Rodima, T. Dansauer, E. B. Rusanov, I. Leito, I. Kaljurand, J. Koppel, V. Pihl, I. Koppel, G. Ovsjannikov, L. Toom, M. Mishima, M. Medebielle, E. Lork, G.-V. Roschenthaler, I. A. Koppel and A. A. Kolomeitsev, *J. Org. Chem.*, 2008, **73**, 2607;

25 L. Lipping, I. Leito, I. Koppel, I. Krossing, D. Himmel and I. A. Koppel, *J. Phys. Chem. A*, 2015, **119**, 735.

26 E. P. L. Hunter and S. G. Lias, *J. Phys. Chem. Ref. Data*, 1998, **27**, 413.

27 a) A. Klamt, *J. Phys. Chem.*, 1995, **99**, 2224; b) A. Klamt, V. Jonas, T. Bürger and J. C. W. Lohrenz, *J. Phys. Chem. A*, 1998, **102**, 5074;

28 A. Klamt and G. Schüürmann, *J. Chem. Soc., Perkin Trans. 2*, 1993, 799.

29 R. Ahlrichs, M. Bär, M. Häser, H. Horn and C. Kölmel, *Chem. Phys. Lett.*, 1989, **162**, 165.

30 a) F. Eckert and A. Klamt, *AIChE J.*, 2002, **48**, 369; b) F. Eckert and A. Klamt, COSMOTherm, COSMOtherm, C3.0, release 1501, COSMOlogic GmbH & Co KG, http://www.cosmologic.de, 2015;

31 a) L. A. Curtiss, K. Raghavachari, P. C. Redfern, V. Rassolov and J. A. Pople, *J. Chem. Phys.*, 1998, **109**, 7764; b) D. Himmel, S. K. Goll, I. Leito and I. Krossing, *Chem. Eur. J.*, 2011, **17**, 5808;
